# EvalDNA: a machine learning-based tool for the comprehensive evaluation of mammalian genome assembly quality

**DOI:** 10.1186/s12859-021-04480-2

**Published:** 2021-11-27

**Authors:** Madolyn L. MacDonald, Kelvin H. Lee

**Affiliations:** 1 Center for Bioinformatics and Computational Biology, University of Delaware, 15 Innovation Way, Newark, 19711 USA; 2 Department of Computer and Information Sciences, University of Delaware, 18 Amstel Ave., Newark, 19716 USA; 3 Delaware Biotechnology Institute, University of Delaware, 15 Innovation Way, Newark, 19711 USA; 4 Department of Chemical and Biomolecular Engineering, University of Delaware, 150 Academy Street, Newark, 19716 USA

**Keywords:** Genome assembly quality, Genome assembly, Machine learning, Chinese hamster, CHO cells

## Abstract

**Background:**

To select the most complete, continuous, and accurate assembly for an organism of interest, comprehensive quality assessment of assemblies is necessary. We present a novel tool, called Evaluation of De Novo Assemblies (EvalDNA), which uses supervised machine learning for the quality scoring of genome assemblies and does not require an existing reference genome for accuracy assessment.

**Results:**

EvalDNA calculates a list of quality metrics from an assembled sequence and applies a model created from supervised machine learning methods to integrate various metrics into a comprehensive quality score. A well-tested, accurate model for scoring mammalian genome sequences is provided as part of EvalDNA. This random forest regression model evaluates an assembled sequence based on continuity, completeness, and accuracy, and was able to explain 86% of the variation in reference-based quality scores within the testing data. EvalDNA was applied to human chromosome 14 assemblies from the GAGE study to rank genome assemblers and to compare EvalDNA to two other quality evaluation tools. In addition, EvalDNA was used to evaluate several genome assemblies of the Chinese hamster genome to help establish a better reference genome for the biopharmaceutical manufacturing community. EvalDNA was also used to assess more recent human assemblies from the QUAST-LG study completed in 2018, and its ability to score bacterial genomes was examined through application on bacterial assemblies from the GAGE-B study.

**Conclusions:**

EvalDNA enables scientists to easily identify the best available genome assembly for their organism of interest without requiring a reference assembly. EvalDNA sets itself apart from other quality assessment tools by producing a quality score that enables direct comparison among assemblies from different species.

**Supplementary Information:**

The online version contains supplementary material available at 10.1186/s12859-021-04480-2.

## Background

Whole genome assemblies are becoming available for an increasing number of organisms due to the reduced time and monetary costs of DNA sequencing. There has been more than a threefold increase in the number of assemblies in NCBI’s RefSeq database since August of 2015 [1] (Table 1). As of September 2021, there was a total of 237,740 assemblies in NCBI RefSeq, consisting of 70,374 unique species. Multiple assemblies are often created for the same species by using different sequencing and/or assembly methods. However, a single genome assembly is typically selected as a reference genome to guide wet-lab and bioinformatics studies. To select the most complete, continuous, and accurate assembly for an organism of interest, comprehensive quality assessment of assemblies is necessary. Researchers should also be aware of any limitations posed by the level of completeness, continuity, and accuracy of their selected reference assembly.

**Table 1:** Assemblies in the NCBI RefSeq Databases in August 2015 [1] and in September 2021 (counts taken on September 8th, 2021)

Taxonomic Group	NCBI RefSeq Aug. 2015	NCBI RefSeq Feb. 2019
Archaea	414	1,154
Bacteria	34,514	223,471
Fungi	167	403
Plants	62	132
Mammals	94	165
All	40,390 (for 12,964 species)	237,740 (for 70,374 species)

The ‘All’ taxonomic group contains viruses and viroids, invertebrates, and protists in addition to the groups listed here. The total assemblies and species counts for Sept. 2021 were determined from the ‘assembly_summary_refseq.txt’ file located at ‘ftp.ncbi.nlm.nih.gov/genomes/ASSEMBLY_REPORTS/’

Genome quality is usually assessed by metrics such as gap percent, N50, and the number of scaffolds that make up the assembly. However, these metrics only reflect the completeness and continuity of an assembly, and not the accuracy. For example, the best assembly is often considered the one with the highest N50, but the N50 metric increases even when contigs are joined incorrectly [2]. One way to evaluate the accuracy of an assembly is to compare it to an existing reference assembly for the organism of interest through a direct sequence comparison. The assembly evaluation tool Quality Assessment Tool for Genome Assemblies (QUAST) [3] and the set of quality evaluation scripts provided by the GAGE study [4] use this method. An updated version of QUAST for larger genome assemblies, QUAST-LG, uses direct sequence comparison as well as k-mer based statistics to evaluate assemblies [5]. However, many de novo assemblies, those built without the use of a reference, do not have a suitable assembly available for comparison.

To overcome this issue, several methods for quality evaluation that do not require an existing reference assembly have been developed. These methods include gene homology methods such as those executed by Core Eukaryotic Genes Mapping Approach (CEGMA) [6] and the more recent Benchmarking Universal Single-Copy Orthologs (BUSCO) [7] programs. The results of these tools reflect the completeness and accuracy of a genome based on expected gene content. However, they only examine the accuracy of well-conserved genes and their copy numbers, rather than the whole genome.

The majority of other reference-independent quality assessment tools use information from mapping sequencing reads back to the genome of interest. Low mapping quality or read coverage can indicate errors in the assembly. Tools using this approach include Amosvalidate [2], ALE [8], FRCbam [9], SuRankCo [10], and REAPR [11]. Amosvalidate was the first automated pipeline for misassembly detection that used read mapping information. However, the pipeline, designed in 2008, uses an older assembly format that is not produced by current assemblers. ALE (Assembly Likelihood Estimator) uses Bayesian statistics to determine the probability of an assembly being correct given a set of reads. The resulting ALE score can be used to compare different assemblies of the same genome, but the authors state that ALE should not be used to compare assemblies across organisms [8]. FRCbam provides a feature response curve for an assembly instead of a numeric score. The curve shows the trade-off between the accuracy and the continuity of the assembly. Similar to ALE, FRCbam can only be used to compare different assemblies of the same organism. SuRankCo uses supervised machine learning where the training data includes metrics from read mapping to rank, rather than score, scaffolds/contigs within a single assembly. REAPR examines the quality of an assembly base-by-base and provides multiple quality metrics derived from read mapping.

Despite the development of these important tools, there is still need for a reference-independent tool that provides a single quality score reflecting the completeness, continuity, and accuracy of an assembly and can be used to compare assemblies from different organisms. Here, we present a novel pipeline called Evaluation of De Novo Assemblies (EvalDNA) to address this need. EvalDNA assists in the modeling of genome quality through supervised machine learning, and uses the subsequent model to estimate a single, comprehensive quality score for a given assembled sequence. The quality score being learned is based on the number of differences in an alignment between a training sequence and its reference, calculated using DNAdiff [12].

EvalDNA calculates completeness and continuity metrics, and uses output from SAMtools [13] and REAPR [11] to generate accuracy metrics. A user-specified model, developed from supervised machine learning, is then used to estimate the quality score using a subset of these metrics. We developed and tested a model for scoring mammalian assemblies which is provided as part of EvalDNA. The resulting scores from EvalDNA can be used to directly compare chromosome sequences within a single assembly, compare multiple genome assemblies from the same organism, and even compare assemblies from different organisms as long as each assembled sequence is scored using the same model.

EvalDNA was applied to human chromosome 14 assemblies from the Genome Assembly Gold-standard Evaluations (GAGE) study [4] to rank genome assemblers and to compare EvalDNA to two other reference-independent quality evaluation tools, ALE and FRCbam. EvalDNA was also applied on more recent, complete human assemblies from the 2018 QUAST-LG study. In addition, EvalDNA was run on several existing Chinese hamster (CH) genome assemblies to compare its results to that of a manual ranking of the assemblies described in Rupp et al. [14] as well as rankings from ALE and FRCbam. This comparison provided insight regarding the performance of EvalDNA on organisms that were not used in the training data and confirmed that EvalDNA can be used to select the highest quality assembly. Scores for each chromosome from the 2018 CH PICR reference genome were also estimated using EvalDNA and compared to chromosomes from the previous CH reference assembly and the reference assemblies for human, mouse, rat, and cow.

Finally, error simulation of PICR chromosomes and scaffolds was done to examine how the EvalDNA score changes as the amount of errors within an assembled sequence increases and to assess EvalDNA’s potential to score scaffolds. The mammalian model’s potential to score plant and bacterial genomes was also briefly examined by applying EvalDNA to several versions of the rice genome assembly and several bacteria genome assemblies from the GAGE-B study [15].

## Implementation

### Overview of the EvalDNA tool

EvalDNA is provided as a Docker instance and is composed of three parts; the Python script to calculate the quality metrics of a given assembly, the scoring model written in R, and the R script to run the model on the calculated quality metrics. Users have two options when using EvalDNA. If the sequence of interest is from a mammalian genome, the user can use EvalDNA with the provided mammalian model. This option would require the user to run the EvalDNA metric calculation pipeline to collect quality metrics for the sequence of interest and then provide the resulting list of metrics to the ‘run model’ script to get the final quality score. Here, we focus mainly on this type of usage.

The second option is to create a new scoring model based on a set of assembled sequences each with a reference sequence. These assembled sequences could be derived from organisms with a high-quality reference genome that are related to the organism of interest. A script is provided to align each of the training sequences to their corresponding reference sequence to get the target quality score. However, scaling of the scores may still be required (see the “Reference-based quality scoring” section). A model would need to be trained on this data and finalized in R, and then loaded into the ‘run model’ script. More on this second type of usage can be found in the EvalDNA documentation.

**Fig. 1:**
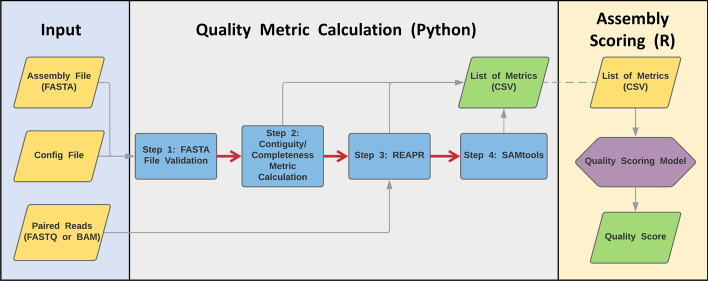
Computational workflow of EvalDNA. EvalDNA requires the assembly of interest in FASTA format, a configuration file, and Illumina paired read data in either FASTQ or BAM file format. EvalDNA first calculates contiguity and completeness metrics, and then calculates accuracy metrics based on the output from running REAPR and SAMtools. This part of EvalDNA produces a list of metrics that will be given to the scoring model (written in R) to estimate the overall quality score for the assembly. The red arrows signify the sequence of steps EvalDNA goes through to calculate various metrics, while the gray arrows signify input and output of data

The steps of the EvalDNA pipeline are shown in Fig. 1. The pipeline requires a configuration file, the sequence(s) of interest in FASTA format, and either a set of paired-end DNA sequencing reads in FASTQ format or a BAM file containing the reads mapped to the sequence(s) of interest. If the raw reads are provided, EvalDNA will run SMALTmap [16], which is the recommended read mapper for REAPR, to map the reads to the provided sequence, creating the BAM file. If the BAM file is provided, EvalDNA can skip the SMALTmap step.

The pipeline calculates the metrics that are used in the mammalian model as well as additional metrics which are still insightful. The selection of metrics used in the model is described in the subsection “Feature selection”. The pipeline first calculates a set of commonly used completeness/contiguity metrics, including percent gaps, N50, and the number of scaffolds/contigs. It then executes REAPR [11], followed by SAMtools stats [13] to calculate various metrics reflecting the accuracy of the given sequence(s). Metrics used in model development are normalized by chromosome length. Once the metrics are calculated and normalized, they are used as input for the R script that applies a user-specified quality model to estimate the assembly quality score.

#### Assembly builds

Chromosomes from the current and previous builds of the rat (Rnor6) and mouse (GRCm38) reference genomes were downloaded from corresponding organisms’ directory on ftp://ftp.ncbi.nlm.nih.gov/genomes/. For instance, mouse chromosome 1 from build37.2 was downloaded from ftp.ncbi.nlm.nih.gov/genomes/M_musculus/ARCHIVE/BUILD.37.2/CHR_01. The FASTA files for each chromosome contained both assembled scaffolds and unplaced scaffolds. Chromosomes from the current human reference genomes (GRCh38) and two assembly builds of another human genome (NA19240) were also used. The NA19240 assembly builds were selected as a training data source because they were built from sequencing reads from a single person. Therefore, differences between reads sequenced from that person’s DNA and the assembly are most likely due to errors in the assembly, rather than true differences among individuals. Deciphering what a sequence difference means in an assembly built from a pool of individuals (such as GRCh38) would be more difficult. Assembly build information can be found in Additional file 1: Table S1.

#### Training data

Training data for the provided mammalian model was collected from rat, mouse, and human assembly builds. Each training instance consists of a set of quality metrics for a single chromosome (see the “Quality metrics” section) and its corresponding quality score (see the “Quality scoring” section). Chromosomes from publicly available assemblies and chromosomes with simulated errors were used, totaling 416 training instances. The full set of training data is provided as Additional file 2.

#### Simulated chromosomes

SINCsimulator [17] was run on a subset of the chromosomes described above to generate errors in the existing chromosomes. Differing levels of single nucleotide polymorphisms, insertions/deletions (indels) and copy number variants were provided to generate chromosomes with differing levels of quality. This resulted in 123 chromosomes with simulated errors. A custom script was used to simulate gaps in 17 chromosomes as well. Both of these steps were done to ensure the model was trained on chromosomes of lower quality than ones that would be submitted to NCBI RefSeq or GenBank.

#### Sequencing reads

20.5 gigabase pairs (Gbp) of Illumina paired-end read sequencing data from ERR319183, ERR316497, ERR316496, and ERR319170 (Bioproject PRJEB2922) was used as input for the metric calculation portion of EvalDNA for all rat assemblies. The insert size was consistent among these runs, ranging from 473 to 475 base pairs (bp), a requirement for REAPR. 25.7 Gbp of Illumina paired-end read sequencing data from ERR1856364 (Bioproject PRJEB19654, insert size 550 bp) [18] was used for the mouse assemblies. 20.2 Gbp of Illumina paired-end read data from the NA19240 human sequencing run SRR2103647 (Bioproject PRJNA288807, insert size 350 bp) was used for the evaluation of both GRCh38 and NA19240 assemblies.

All reads were trimmed using Trim Galore [19] with a quality score cut-off of 26. These reads were used to calculate the accuracy metrics in the training data for the mammalian models. We strongly recommend using at least 10 × coverage of reads with an insert size of approximately 350–550 bp when scoring a novel assembled sequence with the mammalian model to stay consist with the amount and insert size of the reads used to create the training data.

#### Quality metrics

Quality metrics for each training chromosome were calculated using the metric portion of the EvalDNA pipeline. Basic metrics reflecting the completeness and continuity of the chromosome assembly, which include gap percent, N50, N90, scaffold/contig number, and average scaffold length, were collected first.

Several external programs were then run to collect metrics reflecting the accuracy of the assembly. SMALTmap within REAPR maps user-provided reads to the assembly of interest. REAPR then scans the assembly base-by-base identifying possible errors based on the alignment file. The number of bases in each error type, such as bases in clipped reads or low coverage regions, is converted into a percent of the total number of bases to normalize by assembly/chromosome length. Finally, SAMtools is used to calculate the number of read pairs that aligned to the assembly in the expected orientation and distance from one another. These proper read pairs were divided by the number of reads mapped to the assembly to create a proper pair percent metric. Further details on the complete set of metrics and any corresponding normalization can be found in Additional file 1.

#### Reference-based quality scoring

The score for each training instance was calculated based on the NUCmer [12] alignment to the most recent build of the corresponding chromosome. For example, each build of chromosome 1 (query) from the rat genome assembly was aligned to the Rnor6 build chromosome 1 (reference). A score derived from the alignment of bases from the assembly of interest to its reference reflects both the amount and size of misassemblies (translocations, inversions, relocations) and local errors (single base errors and small indels) because the alignment in these areas will either contain inserted gaps or base differences. Contiguity is also reflected by the gaps in the alignment as well, although not as strongly as in metrics such as the corrected N50 or the rankings from FRCbam. The method for calculating the quality score works based on the assumption that more recent builds of an assembly are more accurate. This assumption is supported by the general quality metrics of the assemblies as well as the continuous improvements in DNA sequencing and assembly methods.

For each NUCmer alignment, the number of bases differing between the two sequences were found using DNAdiff. This value was used in the following equation to get the percent of matching (or correct) bases:

$$Percent\,Matching\,Bases=\frac{Length\,of\,Reference-Total\,Differences}{Length\,of\,Reference} * 100$$

Using this method, the self-to-self alignment for each chromosome from the most recent assembly will have a percent of matching bases of 100. However, in practice, not all the chromosomes from the most recent build of an assembly will be of identical quality and neither will chromosomes from different organism assemblies. Therefore, this ‘percent of matching bases’ value cannot directly be used as the score, and instead requires two rounds of scaling; one among the chromosomes in a single assembly (internal scaling) and another to scale between organisms (external scaling).

Internal scale factors were determined by calculating the distance between each chromosome and the ideal set of metrics using Euclidean distance. This ideal set of metrics are metrics that would be produced from a perfectly accurate, continuous, and complete sequence, i.e. gap percent is 0, normalized N50 is 100, percent of error free bases is 100 etc. The best chromosome (i.e. the one with the lowest distance from the ideal chromosome metrics) for each organism kept the score of 100, while the other chromosomes ‘percent of matching bases’ values were scaled based on differences in the distances from the ideal metrics.

A similar process was used to determine the external scale factors. The distances between each organism’s best chromosome and the ideal chromosome metrics were used to determine the best overall chromosome. This best chromosome was chromosome 2 from human NA19240 and kept its score of 100. Again, the distances were used to scale the other organisms’ chromosomes to get the final quality score.

This method allows scores from across species to be compared and also provides context for the EvalDNA score. A chromosome with an EvalDNA quality score higher than 100, for instance, is predicted to be of higher quality than the chromosomes from the human reference assembly as well as chromosomes of NA19240, a more recent Illumina/PacBio hybrid assembly of the human genome.

### Model development

#### Feature selection

To determine which of the 13 quality metrics (features) calculated by EvalDNA were correlated with the target quality scores in the training data, the Pearson correlation (r) between each metric and target quality score was calculated. Quality metrics with a Pearson correlation value between -0.1 and 0.1 were removed before modeling as these metrics only have a very weak linear relationship to the reference-based target quality score. The presence of multicollinearity/redundancy among the metrics was identified by calculating the Pearson correlation value between each pair of metrics. Since multicollinearity among metrics can reduce the accuracy of a model, metrics were further filtered by calculating the joint mutual information using the ‘jmim’ function of the Praznik R package [20] and the percent increase in MSE from the importance function of the randomForest R package [21]. Finally, the ‘regsubsets’ function (exhaustive search) from the R leaps package [22] was then used to create and test linear regression models from subsets of the remaining features (up to 6 metrics). The subset of metrics that created the model with the lowest error and that did not use the links metric was used for the mammalian model.

#### Model training, testing, and selection

Model development and testing was carried out using R statistical software [23]. All models tested were from the Caret R package [24]. The full set of training data was randomly split into two subsets where 80% of the data became the training set and 20% became the testing set. This division resulted in 333 training instances and 83 testing instances.

First, a general linear model using the selected metrics was trained using repeated cross-validation (CV) with 10 folds and 10 repeats. An elastic net model was also trained where cross-validation was used to tune the alpha and lambda hyper-parameters. The models were then applied to the test data to calculate the r-squared and root mean squared error (RMSE) values.

In addition, other types of supervised machine learning models were tested. Models tested included K-Nearest Neighbors (KNN) regression, Random Forest (RF) regression, and Support Vector Machines (SVMs) with linear and polynomial kernels. tenfold cross-validation with 5 repeats was used to tune the KNN model. tenfold cross-validation was used to tune the RF model and fivefold cross-validation was used to tune the SVMs. Each model was applied to the test data to calculate the r-squared and RMSE values. More information about the models is provided in Additional file 1. The model with the lowest RMSE was chosen to be the final model for scoring mammalian assembled sequences.

### EvalDNA pipeline application

#### Application to Chinese hamster genome assemblies

EvalDNA was applied to new assemblies of the Chinese hamster (CH) genome using the mammalian model. Chromosomes from each meta-assembly described in Rupp et al. [14] as well as the previous RefSeq assembly (GCF_000419365.1) [25] and the chromosome-sorted assembly (GCA_000448345.1, CSA) [26] were scored. EvalDNA was also used to score each assembly as a whole with no chromosome separation information provided and including any unplaced contigs.

Illumina reads from SRR954916, SRR954917, and SRR954918 (sequencing project PRJNA167053) [25] were trimmed using a quality cutoff of 26 and a length cutoff of 90 (with the paired option) in Trim Galore. A random subset of trimmed pairs, totaling 20 Gbp, was selected as input for EvalDNA. These sequencing runs were chosen because they had an insert size (∼500 bp) similar to the reads used in the training data.

#### Comparison to other quality evaluation tools

The manual ranking of the Chinese hamster genome assemblies from Rupp et al. [14] were compared to rankings from EvalDNA, FRCbam, and ALE. Normalized EvalDNA scores, scaled between 0 and 1, for the CH genome assemblies were compared to normalized ALE and FRCbam scores. FRCbam and ALE were run using the same Illumina reads used for EvalDNA (described previously). For ALE, the BAM file was created using Bowtie2 [27] (with the ‘-very-sensitive’ parameter) instead of SMALTmap. For an unknown reason, ALE was unable to run when given BAM files created with SMALTmap.

FRCbam was run using the BAM files created with SMALTmap within EvalDNA. FRCbam required tuning for the CE-max and CE-min parameters for each set of chromosomes (i.e. chromosome 8 from all assemblies had the same CE-max and CE-min). Estimation of these parameters was done by first graphing the CE-stats distribution provided by FRCbam without specifying the parameters and then using the 0.95 and 0.05 quantile values from a fitted normal curve as the CE-max and CE-min, respectively. Finally, for each set of chromosomes, the smallest CE-max value was selected to be the CE-max value and the highest CE-min was selected to be the CE-min value. For FRCbam scores, we used the x-value (feature threshold) where the y-value (approximate percent coverage) reached 100% coverage.

#### Quality scoring of GAGE assemblies

The human chromosome 14 assemblies were downloaded from the GAGE datasets website [28]. EvalDNA was run on each assembly to estimate its quality score and subsequently, rank the assemblers. 20.1 Gbp of trimmed paired-end reads from SRR2103647 was given as input. The reads were quality trimmed using Trim Galore with the quality minimum set to 26 to ensure high-quality reads. The EvalDNA results were used to rank the assemblies and the rankings were compared to those reported in the ALE and FRCbam papers.

ALE was run on the assemblers using identical parameters to those stated in the supplementary information for the ALE paper. We were able to replicate their ranking of the assemblers, and additionally scored the CABOG [29] assembly. We also reran ALE with the same parameters, but with a more recent version of Bowtie2 (version 2.3.3.1). For FRCbam scores, we used the x-value where the y-value either reached its maximum or 100 percent depending on which came first. EvalDNA, FRCbam, and ALE scores were normalized to be between 0 and 1 for comparison.

#### Scoring of other assemblies

EvalDNA was run on chromosomes from the reference cow genome (ARS-UCD1.2, GCF_002263795.1). Illumina reads from SRR5753530 were trimmed using a quality cutoff of 26 and a length cutoff of 90 (with the paired option) in Trim Galore. These reads, totaling 20.4 Gbp, were selected to use as input for EvalDNA. Read pairs had an insert size of 600 bp.

EvalDNA was also run on several Japanese rice (Oryza *sativa* ssp. Japonica) assemblies as well as the chromosomes from the reference assembly (Os-Nipponbare-Reference-IRGSP-1.0, GCF_001433935.1). The older versions of rice assemblies examined were GCA_000005425.2 and GCA_000149285.1. All assemblies and the sequencing reads used were from rice of the Nipponbare cultivar. Illumina reads (250 bp long) from SRR547960, SRR547961, SRR547959, SRR547963, and SRR547962 were trimmed using the same parameters for the other organisms. The sequencing reads consisted of 11.5 Gbp and was used as input for EvalDNA. Read pairs had an insert size (450 bp) similar to the reads used in the training data.

In addition, several full human assemblies with published quality and completeness metrics from the QUAST-LG study [5] were evaluated using EvalDNA. Assembly quality was estimated for full genome assemblies from individuals, labelled HG004, built by various assemblers. Contigs and scaffolds less than 3000 bp removed from each assembly before EvalDNA assessment to stay consistent with the QUAST-LG study. Reads, reaching 10 × coverage of the human genome, were randomly sampled from https://ftp-trace.ncbi.nlm.nih.gov/ReferenceSamples/giab/data/AshkenazimTrio/HG004_NA24143_mother/NIST_Illumina_2x250bps/reads/. EvalDNA scores were compared to a variety of continuity, completeness, and accuracy metrics provided in the QUAST-LG study that were calculated using the human genome assembly, GRCh38, as the reference assembly.

#### Error simulation and scoring of PICR chromosomes and scaffolds

Single nucleotide errors were simulated from 5–30%, in increments of 5%, in each chromosome from CH PICR using a custom script. Errors at the same rates were also simulated in scaffolds of various lengths from CH PICR chromosome 1. Errors could be simulated in any location, except for gap regions, across the length of the sequence.

#### EvalDNA application on bacterial assemblies

The GAGE-B study evaluated the quality of several bacterial assemblies [15]. We applied EvalDNA on the scaffold assemblies of three different bacteria species (*R. sphaeroides, M. abscessus,* and *V. cholera*) available on the GAGE-B website [30]. These assemblies were created with a variety of different assembly tools and using HiSeq data provided on the GAGE-B website. Scaffolds less than 500 bp long were removed from the downloaded genome assemblies to replicate what was done in the GAGE-B study. EvalDNA was run, using the available HiSeq data, on the reference assembly for each bacteria as well as the output from seven different assemblers.

EvalDNA was also run on two assembly versions of the *Pseudomonas syringae* pv. actinidiae (Shaanxi_M228 strain) genome (GCF_000344475.2 and GCF_000344475.3). This bacteria was selected because its genome length is more than 5 Mbp, there was available Illumina paired-end sequencing data (SRR8177059), and there were multiple versions of the same assembly where the more recent version is an improved version of the previous. The raw Illumina paired ends were trimmed using Trim Galore with a quality cutoff of 26 and a length cutoff of 50 bp before given as input into EvalDNA. The two assemblies were also compared using NUCmer [12] by first aligning the two assemblies with the more recent version as the reference and the older version as the query. The show-tiling command was then used with the ‘-p’ parameter to convert the query assembly into a pseudomolecule based on overlaps and gaps determined by the alignment. This pseudomolecule was then aligned with NUCmer to the reference for visualization using Dot [31].

## Results

### Training data summary

416 training instances were collected from human, rat, and mouse genome builds. The training data included 276 chromosomes taken directly from publicly available assembly builds, 140 chromosomes with simulated errors, and 17 chromosomes with simulated gaps. Summary statistics (Additional file 1: Table S2) and histograms (Additional file 1: Figure S1) for the metrics in the training data can be found in Additional file 1 as well as the metric definitions.

### Feature selection

The Pearson correlation (r) between each metric and target quality score in the training data was calculated (Table 2). Metrics that were not correlated with quality scores in the training data (-0.1 < r < 0.1) were removed from the model. These included metrics based on the contig number and REAPR’s values for ‘fragment coverage distribution (FCD) error within contigs’ and ‘collapsed repeats’.

**Table 2:** The Pearson correlation coefficient between each metric and the reference-based quality score in the training data that was used to create mammalian model

Quality metric	Pearson correlation
normN50	0.570
gap_perc	− 0.300
prop_pair_perc	0.204
FCD_err_in_contig	− 0.099
FCD_err_over_gap	− 0.295
low_fc_in_contig	− 0.521
low_fc_over_gap	− 0.579
links	− 0.594
clip	− 0.476
coll_repeat	− 0.071
low_read_cov	− 0.402
error_free_bases	0.701
norm_contig_number	0.025

**Fig. 2:**
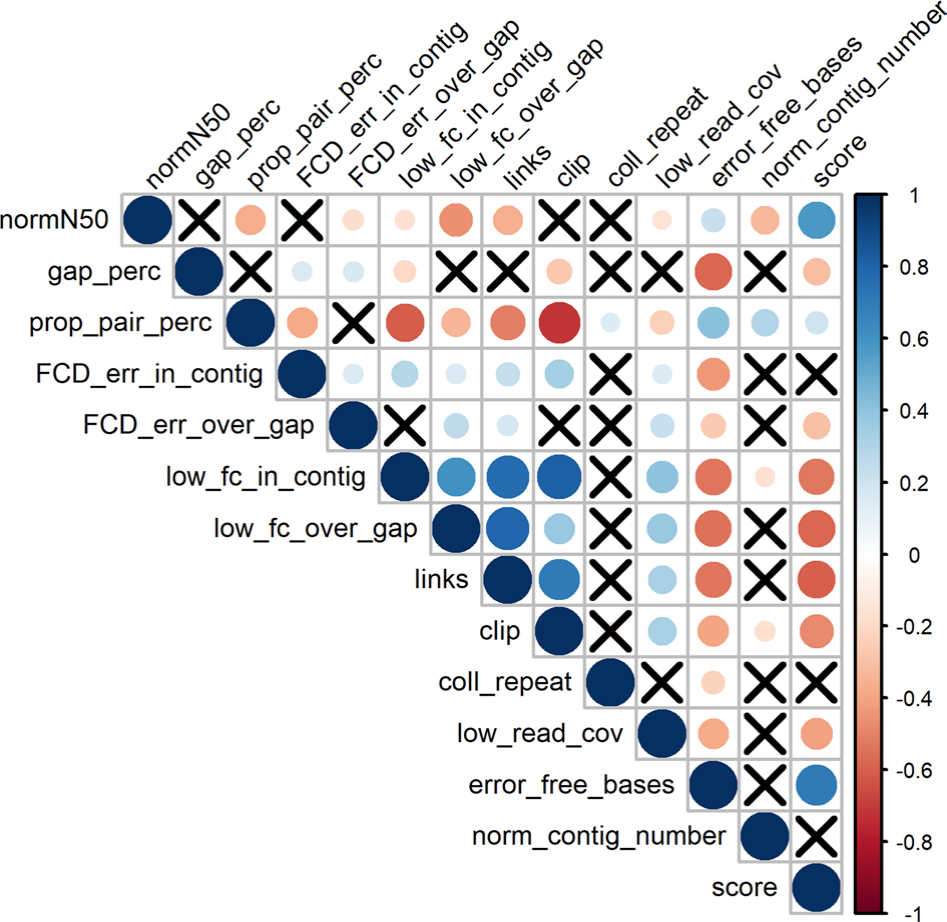
Pearson correlation among all metrics. Cells with an X denote metrics with insignificant correlation. Dark blue represents a stronger positive correlation, while dark red represents a stronger negative correlation

Pearson correlation between each set of metrics showed that more feature selection would be beneficial due to the presence of multicollinearity (Fig. 2). Calculation of joint mutual information among the remaining metrics showed that metrics based on REAPR’s’low read coverage’ and’FCD error over gap’ shared redundant information with other metrics regarding the target score value and could be removed.

The %INCMSE importance metric from the randomForest R package was also examined to see which metrics caused the smallest increase in mean squared error (MSE) when replaced by a randomly permuted variable in a random forest regression model. This method suggested that the ‘proper pair percent’ and ‘FCD error over gap’ metrics could be removed. Results of these feature selection methods are provided in Additional file 1: Tables S3 and S4.

Subsets of the remaining features (normN50, gap_perc, clip, error_free_bases, links, low_fc_over_gap, low_fc_in_contig) were used to generate linear regression models to see which metrics produce high performing models (Additional file 1: Figure S2). The six best performing models all produced an r-squared of 0.74 with the top two models having the smallest residual sum of squares values. NormN50, gap_perc, clip, error_free_bases, low_fc_over_gap, and low_fc_in_contig were chosen as the metrics for subsequent modeling of the quality score. Low_fc_in_contig was chosen rather than links because while links has a negative correlation with quality score (Table 2), it is given a positive weight within the linear regression model. This suggests that there may still be concerns with multicollinearity when using the links metric.

It is important to note that the metrics selected for the model do allow a perfect score to be calculated for a portion of an assembly, either a contig, scaffold, or chromosome. EvalDNA with this model would assign as a score of 100 when NormN50 is 100, gap_perc is 0, clip is 0, error_free_bases is 100, low_fc_over_gap is 0, and low_fc_in_contig is 0, which could occur for any sized assembly which had no gaps or errors. Therefore, the user should be aware what they are scoring (a full assembly, a chromosome, or a smaller piece of an assembly). If a user is interested in scoring only full assemblies, a model including a metric reflecting the percent of aligned sequencing reads could be used instead of the provided model.

### Model selection

RMSE and r-squared values for each possible model type were calculated (Table 3). These values reflect each model’s performance on the test set. More specifically, the r-squared values reflect the proportion of the reference-based quality score that can be explained by each model, while the RMSE values reflect the differences between the reference-based quality scores and those predicted by each model.

**Table 3:** The r-squared and RMSE values for each type of regression model that was tested to select the best performing model (highlighted in bold) to be the mammalian model

Regression model	RMSE	R-squared
General linear	16.413	0.775
Elastic net	16.520	0.773
K-nearest neighbors	13.615	0.840
**Random forest**	**12.697**	**0.860**
SVM (linear)	17.190	0.774
SVM (polynomial)	14.363	0.843

**Fig. 3:**
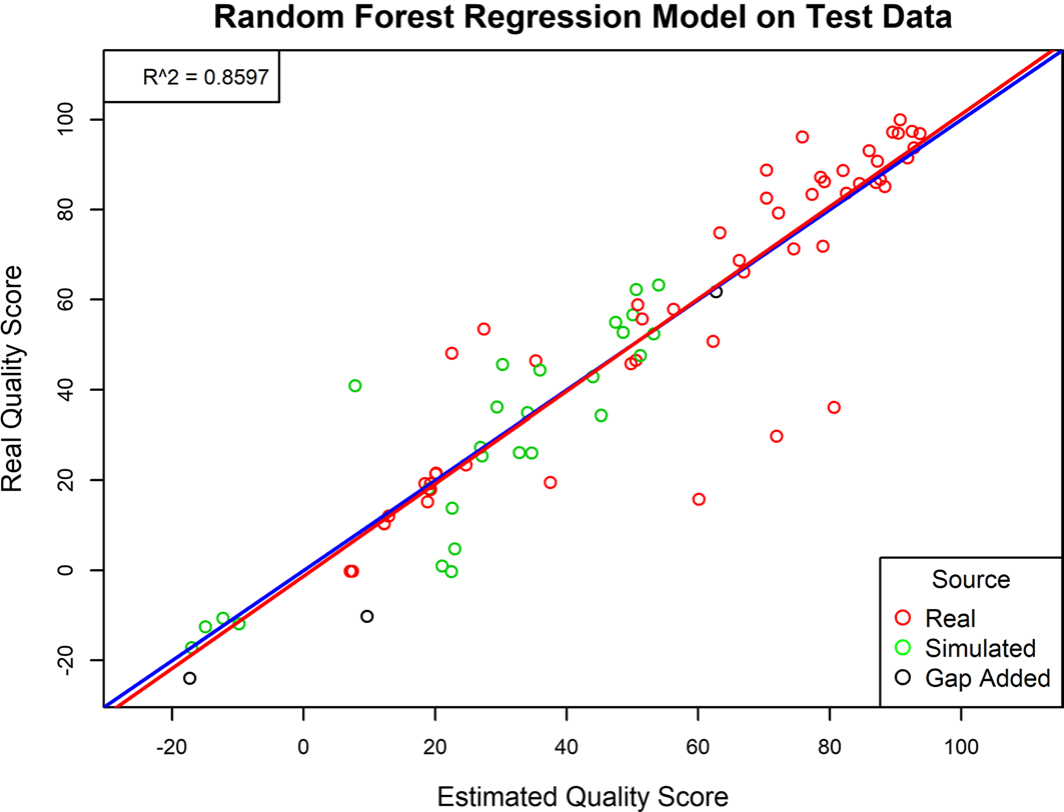
Performance of the random forest regression model on test data. Estimated quality scores for the test instances are plotted against the reference-based quality scores of the test instances. A 100% accurate model would produce the blue line with an r-squared equal to 1. The line of best fit for the plotted data is shown as the red line and has an r-squared of 0.8597

The best performing model was random forest regression with 500 trees and an mtry value (number of variables tested at each split) of 2. This model produced an RMSE of 12.697 and an r-squared of 0.860 when applied to the testing data (Fig. 3). The random forest model was retrained on the full data to develop the final model that would be used to predict the quality scores of mammalian genome assemblies.

Once the final model was selected, we wanted to confirm that the source of the assembled sequences in the training set did not impact the ability of the model to predict quality scores. There were no clear patterns regarding the residuals of the scores versus organism source (data point shapes) or regarding the residuals of the scores versus the generation method of the chromosomes i.e. if they were real, simulated, or had gaps added (data point colors) (Additional file 1: Figure S3). This observation suggests that the model’s ability to predict quality scores of instances within the training/testing data was not impacted by organism or generation methods.

### Applications of EvalDNA and comparison to other quality evaluation tools

#### Evaluating assemblers used in the GAGE study

**Fig. 4:**
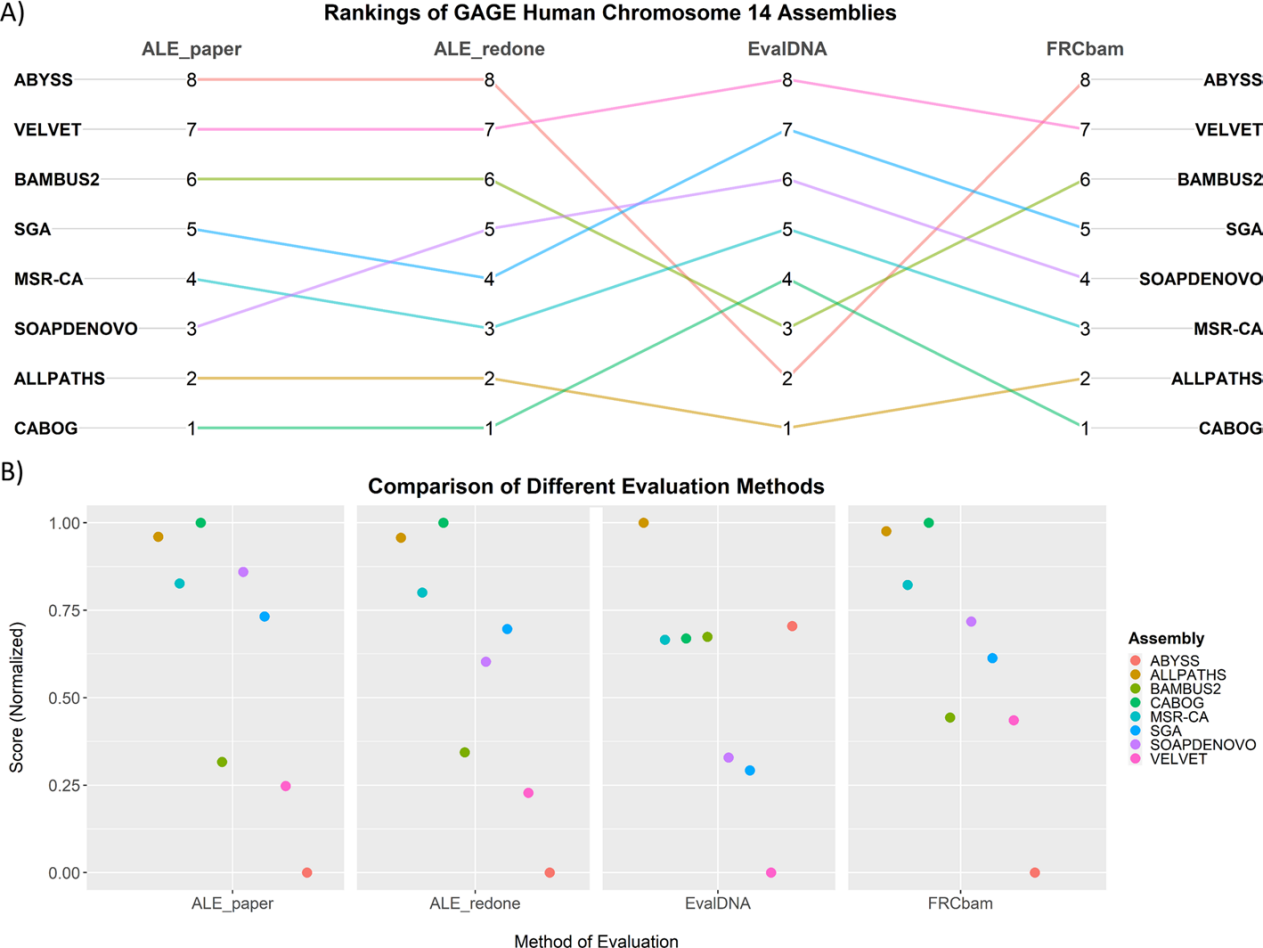
Comparison of quality evaluation methods on human chromosome 14 assemblies (from the GAGE study). **A** The EvalDNA ranking of assemblers used to build the human chromosome 14 assembly are compared to the rankings from ALE and FRCbam. The highest quality assembly is given a rank of 1. **B** EvalDNA and ALE scores for the human chromosome 14 assemblies were normalized (scaled to be between [0, 1]). ALE paper scores were calculated using the same parameters and version of Bowtie described in Clark et al. The ALE redone scores were calculated with an updated version of Bowtie. FRCbam normalized scores were derived using the x-value (feature threshold) where the y-value (percent approximate coverage) was maximum for each FRCbam curve

EvalDNA with the mammalian model was used to score and rank the different assemblies of human chromosome 14 from the GAGE study [4, 28]. The rankings were compared to rankings generated during the original benchmarking tests for ALE and FRCbam as well as the ranking generated by running ALE with an updated version of Bowtie (Fig. 4A). Normalized EvalDNA scores (scaled to be between [0,1]) were compared to the two sets of normalized ALE scores and the normalized FRCbam scores (Fig. 4B).

EvalDNA and FRCbam selected ALLPATHS-LG [32] as the best assembler, while the ALE runs ranked ALLPATHS-LG as the second best with the CABOG assembler [29] ranking first. EvalDNA ranked the assembly produced by Velvet [33] as the lowest quality assembly, which is not surprising since it is made up of approximately 45% gaps. The Velvet assembly was ranked second to last by the ALE runs and third to last by FRCbam.

One key difference among the rankings is that EvalDNA ranked the ABySS [34] assembly much higher (second place) than either ALE or FRCbam (last place). ALE and FRCbam most likely ranked ABySS the lowest because the assembly is highly fragmented. However, the ABySS assembly is also one of the more accurate assemblies with fewer scaffold misjoins, inversions, relocations, and indels than the other assemblies [15]. ABySS also has a very low gap percent (0.53%). This observation suggests that EvalDNA’s mammalian model may value accuracy and completeness (in regards to the lack of gaps) over continuity more so than ALE or FRCbam. In addition, examining the normalized EvalDNA scores does show that ABySS, while second in the ranking, scored only slightly better than the CABOG, MSR-CA [35], and BAMBUS2 [36] assemblers.

#### Scoring of Chinese hamster assemblies for reference assembly selection

In 2018, four assemblies for the Chinese hamster (CH) were built using PacBio sequencing data and existing Illumina data. Manual ranking of these new assemblies as well as two Illumina-only assemblies from 2013 was completed to select the best reference genome for CH and Chinese hamster ovary (CHO) cells [14]. The two Illumina-only assemblies included the 2013 CH RefSeq assembly [25] and the 2013 chromosome sorted assembly (CSA) [26]. EvalDNA results were compared to this ranking to evaluate its performance on real assemblies outside of those used in the training data and if it could be used to select the best assembly to be the new reference genome. EvalDNA with the mammalian model was used to score the six different CH assembly versions (Table 4) as well as each chromosome from the assemblies (Table 5). Scaffolds and contigs were assigned to chromosomes based on the coverage of reads mapped from each of the CSA chromosomes. For CSA, sequencing was done on chromosomes after they were individually isolated using flow cytometry. However, chromosomes 9 and 10 could not be separated due to their size similarity [26]. Therefore, for each assembly, scaffolds could be assigned to chromosomes 9 and 10, but not separately, and these chromosomes together are given a single score. The full CH assemblies were also assessed by FRCbam and ALE.

**Table 4:** The EvalDNA quality scores for each Chinese hamster genome assembly

Assembly	Mammalian model	Model without N50
PICR (2018 RefSeq)	70.22	88.47
PIRC	70.20	88.41
IPCR	57.56	59.29
IPRC	57.57	59.22
2013 RefSeq	58.72	64.35
CSA	43.21	40.60

**Table 5:** The EvalDNA quality scores for each chromosome of the Chinese hamster genome assemblies

Chromosome	PICR	PIRC	IPCR	IPRC	RefSeq	CSA
1	**72.04**	71.91	60.11	59.26	59.58	41.51
2	71.00	**71.20**	58.76	57.37	56.92	49.90
3	65.37	**65.42**	55.56	54.51	55.21	39.55
4	**68.75**	68.63	54.71	56.01	56.15	42.49
5	68.02	**68.21**	40.01	63.13	56.38	47.48
6	68.21	**68.24**	62.77	56.70	56.40	50.27
7	**68.95**	68.66	55.89	54.77	55.80	46.44
8	**63.99**	63.84	52.21	56.45	54.47	52.83
9,10	48.31	**52.32**	39.53	51.92	47.62	47.27
X	**53.30**	52.51	49.30	53.14	48.72	30.44

The highest score for each chromosome is highlighted in bold

**Fig. 5:**
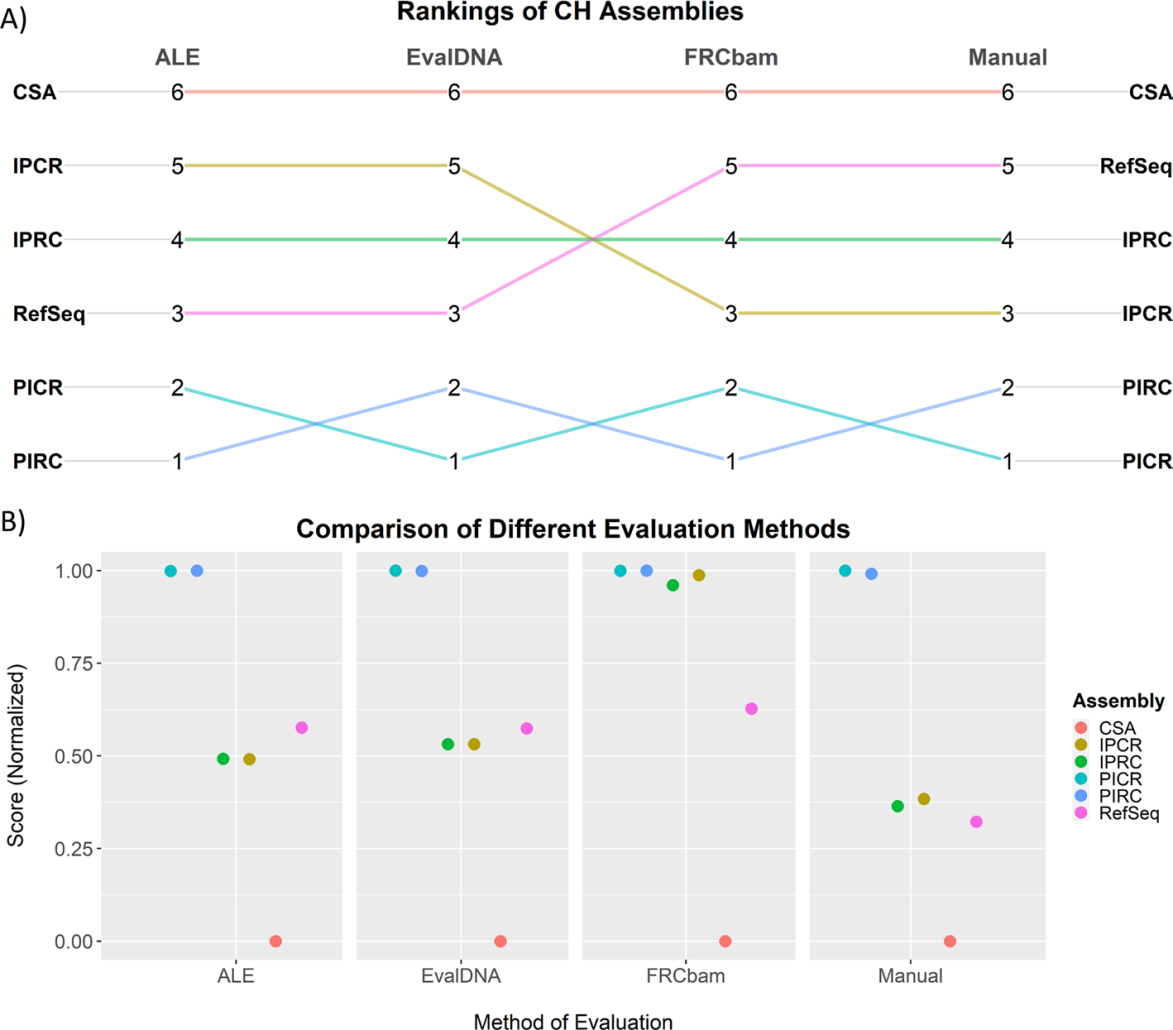
Comparison of quality evaluation methods on Chinese hamster genome assemblies. **A** Comparison of the EvalDNA ranking of the multiple CH genome assemblies to a manual ranking, and rankings from ALE and FRCbam. The highest quality assembly is given a rank of 1. **B** EvalDNA and ALE scores for the CH assemblies as well as the rankings from Rupp et al. and FRCbam were normalized (scaled to be between [0, 1]). FRCbam normalized scores were derived using the x-value (feature threshold) where the y-value (percent approximate coverage) was maximum for each FRCbam curve

EvalDNA and the manual ranking selected PICR as the CH assembly with the highest overall quality (Fig. 5A), with PIRC a close second. FRCbam ranked PICR and PIRC as the highest, but the curves were too close to distinguish between them (Additional file 1: Figure S4). All four evaluation methods agreed that CSA was of the poorest quality. However, EvalDNA and ALE both scored RefSeq higher than IPCR and IPRC, while the manual ranking and FRCbam had this order switched. Examining the ALE and EvalDNA normalized scores more closely (Fig. 5B) show that these three assemblies are very similar regarding quality (within 0.05 normalized units). The difference in quality may be too small for EvalDNA to meaningfully distinguish between these assemblies.

The accuracy of EvalDNA scores and ranking of CH assemblies was also confirmed by calculating the number of differences between each CH assembly and the ‘reference’ genome (PICR). This method allows each assembly to get a score, calculated the same way the training instances were scored with the exception of not being scaled (see “Quality Scoring” section in Methods). The difference between each assembly’s score and a score of 100 (PICR’s score from aligning PICR to itself) should be similar to the difference between the corresponding assembly’s EvalDNA score and PICR’s EvalDNA score. The differences were indeed similar (Additional file 1: Table S5), confirming that EvalDNA can be used to accurately evaluate assemblies from organisms that were not used in the training set.

#### Comparing CH assembly quality to other organism reference assemblies

**Fig. 6:**
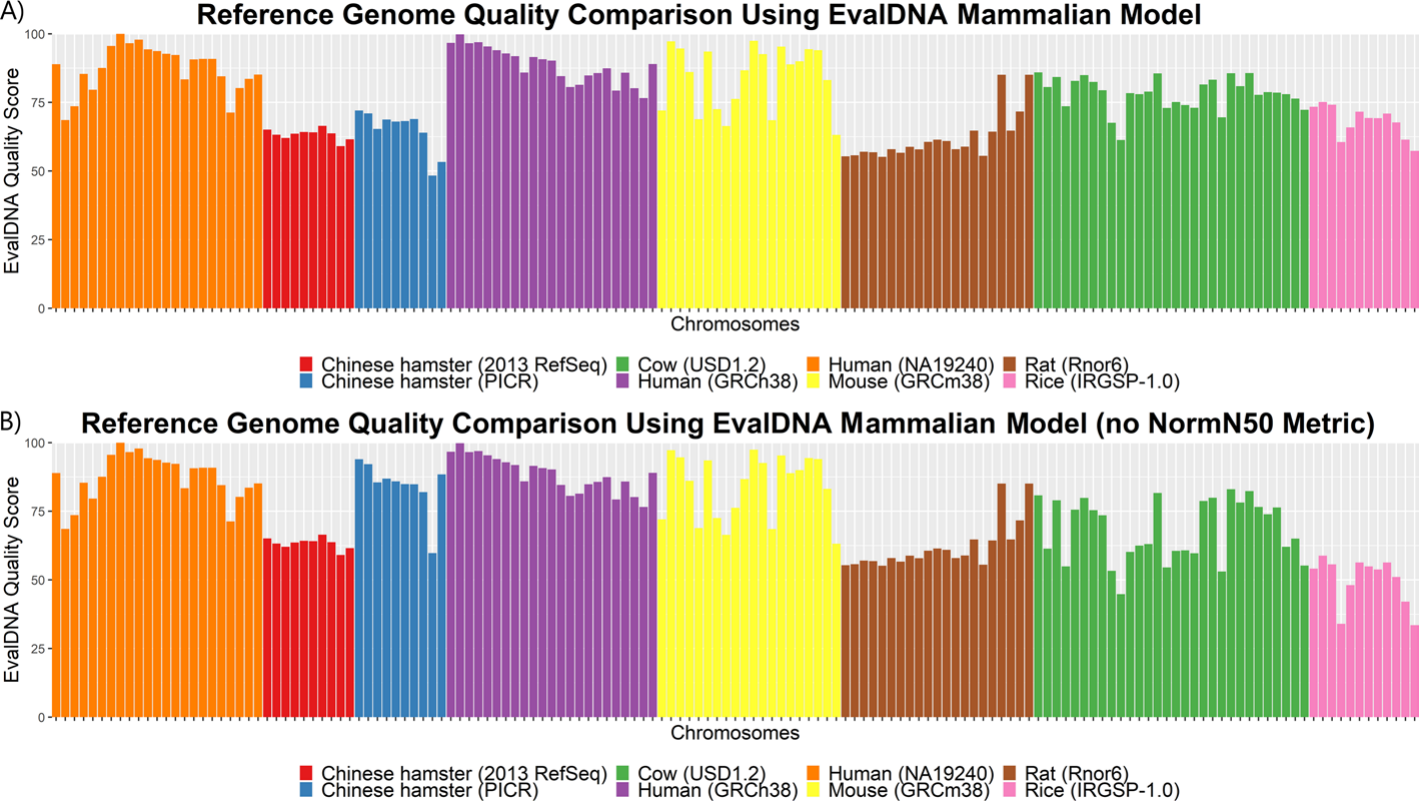
EvalDNA quality scores for chromosomes from various genome assemblies. **A** EvalDNA quality scores for chromosomes from CH PICR, CH 2013 RefSeq, and the mouse, rat, human, cow, and rice reference genome assemblies. **B** EvalDNA quality scores for the same chromosomes but calculated using a model that does not include the normalized N50 metric

The PICR assembly was selected by the community to be the new Chinese hamster reference assembly (GCF_003668045.1) [14]. EvalDNA scores for the PICR chromosomes were compared to scores from the 2013 CH RefSeq assembly and the reference assemblies for human (GCF_000001405.38), mouse (GCF_000001635.2), rat (GCF_000001895.5), and cow (GCF_002263795.1). The majority of the PICR CH assembly chromosomes are of higher quality than those of the 2013 CH RefSeq assembly and the rat reference assembly (Fig. 6A). Several chromosomes also scored as high as those from the mouse reference assembly.

EvalDNA was also run on each chromosome from the rice (*Oryza sativa*) reference genome (GCF_001433935.1) (Fig. 6A). While the model was trained using mammalian data, the results of EvalDNA with this model on rice also seem reasonable. Two older versions of the rice assembly, Build4.0 (GCA_000005425.2) and OrySat_Sep2003 (GCA_000149285.1), were scored. Build4.0 scored within 1 unit of the most recent version, while OrySat_Sep2003 scored significantly lower (more than 30 units). The similar scores between Build4.0 and the most recent reference is not surprising because the accuracy of Build4.0 was already high with an error rate estimated to be less than one per 10,000 nucleotides and possibly as low as 0.15 errors per 10,000 nucleotides [37]. Results of the rice assemblies are given in Additional file 1: Table S6.

The scores allow comparison of assemblies across organisms in regards to continuity, completeness, and accuracy. Changing the model to only examine a subset of these categories can give more specific insight into where an assembly excels or needs improvement. For instance, we scored the chromosomes with a different random forest regression model which does not include the normalized N50 metric (Fig. 6B). This model, described in Additional file 1, enables comparisons across organisms based on completeness and accuracy only. The model shows a large increase in the accuracy and completeness of the 2018 CH PICR reference assembly over the 2013 CH RefSeq assembly. In addition, because each chromosome of the cow and rice assemblies contains just a single scaffold, the original model scored each chromosome from these organisms much higher than the model that does not use the normalized N50 metric (Fig. 6). The disparity among the scores predicted between these two models does confirm that scores from different models are not directly comparable.

#### EvalDNA scores correlate with error simulation rates, but not linearly

To examine how changes in the amounts of errors within an assembly affect the EvalDNA score, we ran EvalDNA on versions of the CH PICR chromosomes which contained varying amounts of randomly generated single nucleotide errors. Single nucleotide changes were simulated from 5 to 30% in increments of 5%.

**Fig. 7:**
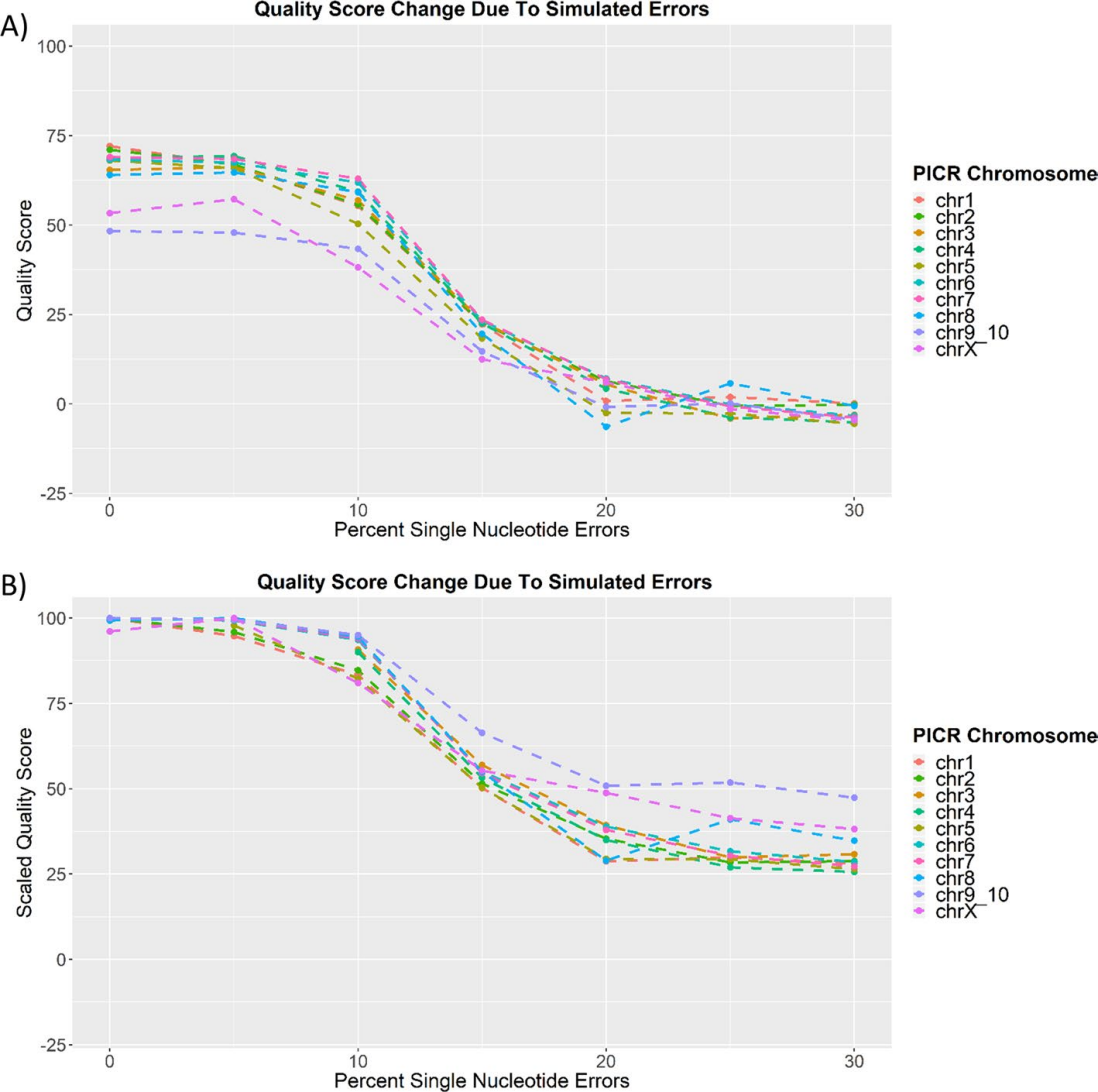
Impact of error rates on the EvalDNA quality scores of CH PICR chromosomes. **A** Changes in EvalDNA quality scores due to simulation errors. **B** Changes in scaled EvalDNA quality scores due to simulation errors. Scores were scaled so that the maximum score for a chromosome became 100

Each simulated chromosome was scored by EvalDNA (Fig. 7A). Similar trends across all chromosomes are seen, and the scores do not linearly decrease as the amount of errors increase. On average, the quality score decreases slightly (1 unit) between a 0% error rate and a 5% error rate and then decreases an average of 10 units between 5 and 10% error rates. An even larger score decrease (average of 34 units) occurs as the simulated error rates change from 10 to 15%. The scores decrease an average of 17 units from 15 to 20%, 3 units from 20 to 25%, and 2 units from 25 to 30%.

Assessment of the EvalDNA scores with respect to error rates alone is difficult because none of the PICR chromosomes are perfectly accurate, complete, and continuous before error simulation. The chromosomes with 10% simulated error rate have scores anywhere from 35 to 65 depending on the continuity and completeness of the chromosome. However, a near perfect chromosome or assembly will have a score above 100 and insights can be gained from scaling all the scores so that maximum score for each chromosome is 100 (Fig. 7B). From scaling, we can see that a perfectly complete and continuous chromosome with a score around 89 corresponds with an error rate of approximately 10%. This means that a chromosome or assembly that is not fully complete and continuous with a score of 89 or above will have a percent error rate lower than 10%. Since most mammalian assemblies are far from being fully continuous and complete, a score of 89 will often mean an error rate of much lower than 10%. Even the chromosomes from the current human reference genome assembly (GRCh38) in the training set have scores ranging from approximately 85 to 100, and GRCh38 has an estimated error rate of 1 in 100,000 bases (0.001%) [38]. Recommended guidelines for how to categorize an assembly based on the reference-based quality scores from the training data are provided in Additional file 1: Figure S5.

#### EvalDNA application on scaffolds

Varying levels of single nucleotide errors were randomly generated in several scaffolds from PICR chromosome 1 to examine how well EvalDNA with the mammalian model performs on scaffolds. To minimize false mapping, EvalDNA was run using only reads that mapped to the original scaffold with an identity of 0.75 (at least 75% of the bases needed to match).

**Fig. 8:**
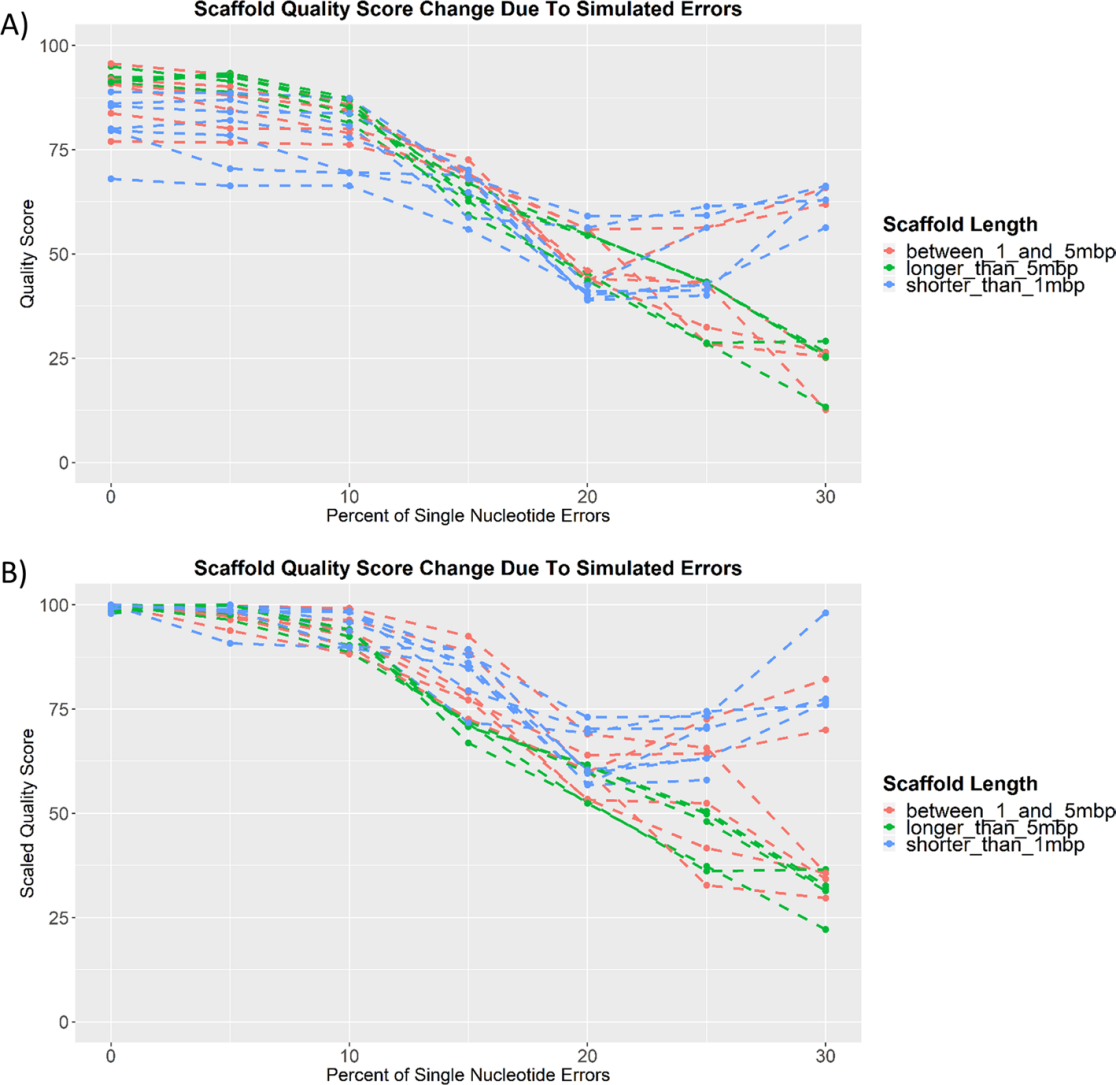
Impact of error rates on the EvalDNA quality scores of CH PICR scaffolds. **A** Changes in EvalDNA quality scores due to simulation errors. **B** Changes in scaled EvalDNA quality scores due to simulation errors. Scores were scaled so that the maximum score for each scaffold became 100

The error simulation results suggest that EvalDNA’s ability to estimate quality scores for scaffolds depends on the amount of errors and the scaffold length (Fig. 8). The score decreases in a similar manner as the chromosomes did for all length scaffolds with 0–10% errors simulated. As the percent of errors increases beyond 10%, the impact of length on the scores becomes apparent. The scores show the expected decreasing trend for scaffolds longer than 5 Mbp, although at a slower rate than the chromosome scores. The expected decreasing trend is not observed for scaffolds shorter than 1 Mbp and for only some of the scaffolds between 1 and 5 Mbp long. Therefore, a model specifically trained on scaffolds in these length ranges would be beneficial for short scaffold scoring.

#### EvalDNA application on human assemblies from the QUAST-LG study

EvalDNA was applied to four human draft genome assemblies for the individual labelled HG004 that were previously evaluated in the QUAST-LG paper [5]. The four draft assemblies were all significantly more fragmented than any of reference assemblies scored by EvalDNA for the CH genome assembly comparison and also had a large number of misassemblies when compared the human reference assembly, GRCh38 [5]. EvalDNA appropriately produced low scores for all the draft assemblies (Additional file 1: Table S7). SOAPdenovo2 assembly was the most fragmented with 55,725 contigs (10 × more fragmented than the other draft assemblies), had the lowest N50 (258,443 bp), and had a large number of misassemblies (670). As expected, EvalDNA gave the SOAPdenovo2 assembly the lowest score of the four draft assemblies (− 11.58). The ABySS2, Discovar, and UpperBound assemblies scored similarly at 8.78, 8.42, and 9.35 respectively. The UpperBound assembly, which was created to be the theoretical assembly optimum given the set of reads and the GRCh38 reference assembly [5], was surprisingly not much higher than the other two draft assemblies despite this not having any misassemblies when compared to GRCh38. However, the UpperBound assembly was still highly fragmented (4,958 contigs) which caused the EvalDNA score to remain low.

#### EvalDNA application on bacterial assemblies from the GAGE-B study

EvalDNA correctly scored the reference genome assemblies of the bacteria from the GAGE-B study higher than that of the non-reference assemblies (Table 6). However, in general, EvalDNA had difficulty distinguishing between the outputs of the different assemblers used in the GAGE-B study. The scores from EvalDNA for each bacteria across the different assemblies were all within five units of each other. There was also little correlation between the EvalDNA score and the corrected N50 values reported in the GAGE-B study, except for *Vibrio cholerae* which showed the expected positive trend with a Pearson correlation coefficient of 0.58. However, when the EvalDNA score and corrected N50 from the reference assemblies are considered, there is a strong positive correlation (r > 0.95) for all bacteria (Additional file 1: Figure S6). All three bacteria also showed the expected negative trend, with Pearson correlation coefficients from -0.52 to -0.75, when plotting the EvalDNA score versus the number of large (> 1 kb) errors reported in the GAGE-B study for each assembly (Additional file 1: Figure S7). It is important to note that EvalDNA scores could accurately differ in order, from the corrected N50 values or large errors, because the EvalDNA score includes more quality metrics such as the amount and size of gaps and local errors.

**Table 6:** The EvalDNA quality scores for each bacterial assembly evaluated from the GAGE-B study

Bacteria	Reference	Abyss	Cabog	MSR-CA	SOAP	SGA	Spades2.3	Velvet
M.abscessus	**83.83**	67.16	71.62	70.18	69.91	70.79	70.59	71.77
R.sphaeroides	**86.42**	73.33	74.54	74.32	76.56	75.05	72.6	74.78
V.cholerae	**85.4**	72.18	69.69	69.78	70.23	70.57	72.15	71.25

The highest score for each chromosome is highlighted in bold

EvalDNA scores calculated for the two assembly versions of the *Pseudomonas syringae* genome reflected the improvement between assembly versions with the most recent version scoring an 82.54 and the older version scoring a 74.44. The earlier version of the assembly consisted of 419 contigs and had an N50 of 38,960 bp, while the more recent version was a completed assembly, consisting of two contigs (1 for a chromosome, and 1 for a plasmid). While the earlier version is significantly more fragmented than the completed version, alignment using NUCmer [12] showed high percent similarity and coverage between the contigs in the earlier version and the two completed sequences in the more recent version (Additional file 1: Figure S8). This high accuracy is most likely the reason EvalDNA still assigned a good score (~ 70) to the older version.

#### Runtimes and memory usage for EvalDNA

EvalDNA runtimes are dependent on the size of the input assemblies and the read FASTQ files. Runtimes are shortened if the reads are already mapped to the assembly and a BAM file is provided. On average, EvalDNA takes several days to run on full mammalian assemblies with 16–24 processors and takes about a day for a mammalian chromosome (Table 7). The default Docker memory limit of 256 Mb was sufficient for scoring mammalian assemblies. For the bacterial assemblies tested, EvalDNA took less than an hour to score four assemblies (each about 5 Mbp long) and had a maximum memory requirement of about 75 Mb.

**Table 7:** EvalDNA runtimes on mammalian full and chromosomal assemblies

Assembly	Time	Number of processors	Assembly length (Mbp)	Number of reads (millions)
CH PICR	1 day 11 h	16	2,368.91	179.59
CH PICR Chr8	11 h. 59 min	16	96.66	179.59
CH PICR Chr10	8 h. 13 min	16	32.58	179.59
CH PICR Chr4	16 h. 35 min	16	231.54	179.59
UpperBound Human (from QUAST-LG)	9 days 10 h	24	2,916.50	159.69

## Discussion

Here, we presented a novel pipeline, called EvalDNA, for genome quality assessment that does not require a reference genome. We also developed a model, trained on mammalian assembly data, to be used within EvalDNA. The model evaluates an assembly based on completeness, continuity, and accuracy by using the normN50, gap_perc, clip, error_free_bases, low_fc_over_gap, and low_fc_in_contig metrics.

The EvalDNA pipeline with this mammalian model was able to accurately estimate the quality scores of Chinese hamster genome assemblies and enabled the comparison of CH chromosomes to those from other organisms’ reference genome assemblies. EvalDNA can also be used to examine the output of different assemblers as demonstrated on the human chromosome 14 data from the GAGE study and on complete human assemblies from the QUAST-LG study.

While EvalDNA with the mammalian model appeared to weigh accuracy over continuity more so than existing tools such as ALE and FRCbam, the model without the normalized N50 metric can be used to score assemblies completely independent of continuity if needed. This model may be useful for situations such as genome annotation, where the accuracy and completeness of an assembly is more important than continuity. A model without the normalized N50 metric could also be useful when comparing chromosomes from different assemblies where the method of how the scaffolds/contigs were assigned to a chromosome may differ and may impact the quality score. Another use case that could benefit from a slightly different model than the one provided would be if the user was interested in scoring and comparing only whole assemblies and wanted to consider the completeness of each assembly. As noted previously, the provided model can assign a perfect score to a portion of an assembly i.e. a chromosome. Instead, to avoid this perfect score assignment to segments of an assembly, the user could use a model that includes a metric that reflects the percent of aligned reads. This model would not assign a perfect score to a shorter piece of an assembly as 100% of the reads should only align if the assembly is complete or almost complete. However, it is important to note that scores from different models should not be directly compared.

### Benefits of a comparable genome assembly score

EvalDNA provides the ability to assign a comprehensive quality score to all assemblies and all chromosomes made available online. A researcher would be able to easily select the best available assembly for their organism of interest from viewing these scores, and even choose the best version of a specific chromosome. More confidence could also be given to findings derived from a high scoring reference genome than findings from a lower scoring reference genome.

The assigned quality score would also be comparable across organisms scored by the same model. The scores would provide insight into how a chosen assembly compares to “gold-standard” genomes, such as the human reference assembly, in terms of overall quality. Because EvalDNA can only be used to compare assemblies from different organisms if the assemblies were scored using the same model, the applicability of the mammalian model across all species should be examined in more depth. Initial results on the rice assembly do suggest that the mammalian model could work to assess plant genome assemblies, but more study is needed. The results on the bacterial assemblies suggest that EvalDNA can correctly distinguish between bacteria assemblies (with lengths of approximately 5 Mbp) with significantly different levels of quality.

### Applying EvalDNA to scaffolds

The principles used within EvalDNA can be applied to scaffolds as well. However, the mammalian model has been created specifically for whole and chromosome level assemblies. The training data for the mammalian model was generated using the mapping defaults of SMALTmap within REAPR. This only required reads to have at least 50% of bases match the reference to be mapped. For scaffolds, this threshold causes a significant amount of incorrect read mapping as reads from anywhere in the genome could map to the scaffold and therefore, a higher mapping stringency is needed. Initial results of the mammalian model on scaffolds longer than 5 Mbp seem promising, but did require increasing the mapping stringency to 0.75 (75% bases need to map). Therefore, while the model can be applied to scaffolds longer than 5 Mbp if a higher percent mapping threshold is specified, the resulting quality score will not necessarily be directly comparable to the scores of chromosomes or whole genome assemblies.

### Model improvement

The current model on average predicts the score within 13 units of the real score and is able to explain 86% of the variation in quality scores. Therefore, there is potential for model improvement. First, increasing the number of chromosome instances in the training set would help the model become more precise. In addition, the model may benefit from the addition of quality metrics not tested here. The new metrics may be able to capture the remaining 14% of the score quality not captured by the current model.

### Long-read sequencing

Currently, high-quality paired-end Illumina reads are required to use EvalDNA. A future goal is to extend EvalDNA to use longer reads, such as those from PacBio or Oxford Nanopore sequencing, to assess accuracy either alone or along with Illumina data. This improvement will require the development of metrics that reflect the accuracy of an assembly based on the mapping of long reads. Possible metrics could include the percent of high-quality mapped long reads or the total length of structural variants identified from the long read mapping.

## Conclusions

We developed and tested a novel pipeline, called EvalDNA, for the evaluation of genome assembly quality that does not require a reference genome. A model, which can be used within the pipeline, was created using supervised machine learning. The model examines the accuracy, continuity, and completeness of either an assembled genome or chromosome, and was able to predict reference-based quality scores of assemblies with an accuracy of approximately 86%.

EvalDNA will allow scientists working with multiple genome assembly versions to identify the most appropriate one to be their reference genome, as well as examine which chromosomes may need to be improved. EvalDNA also enables quality comparison against other organism assemblies, such as high-quality reference human and mouse assemblies.

## Availability and requirements

Project name: EvalDNA.

Project home page: https://github.com/bioinfoMMS/EvalDNA.

Operating system(s): Platform independent.

Programming language: Python v2.7.13, R statistical software v3.5.1 or later.

Other requirements: Docker.

License: GNU GPLv3.

No restrictions to use by non-academics.

## Supplementary Information

**Additional file 1**: Additional metric descriptions, feature selection results, tables and figures.

**Additional file 2**: A text file in comma-separated value (CSV) format, containing the full training data set used to create the mammalian model.

## Data Availability

The training data set generated and used to develop the models described in this article is included as Additional file 2. The genome assemblies and paired-end read data are available from NCBI Assembly/Genome and the Sequence Read Archive (SRA) respectively. Assemblies used were human (GCF _000001405.38, GCA_001524155.4, GCA_001524155.1), mouse (GCF_000001635.2 and builds 30, 33.1, 34.1, 35.1, 36.1, 37.2), rat (GCF_000001895.5, GCF_000001895.3 and builds 2.1, 3.1, 4.1), Chinese hamster (GCF_003668045.1, GCF_000419365.1 and GCA_000448345.1), cow (GCF_002263795.1), Japanese rice (GCF_001433935.1, GCA_000005425.2, GCA_000149285.1), *Pseudomonas syringae* (GCF_000344475.2, GCF_000344475.3). The SRA datasets used were mouse (ERR1856364), rat (ERR319183, ERR316497, ERR316496, and ERR319170), human (SRR2103647), Chinese hamster (SRR954916, SRR954917, and SRR954918), cow (SRR5753530), Japanese rice (SRR547960, SRR547961, SRR547959, SRR547963, and SRR547962), and *Pseudomonas syringae* (SRR8177059). The human chromosome 14 GAGE assemblies were downloaded from http://gage.cbcb.umd.edu/data/index.html. The full human assemblies from the QUAST-LG paper were downloaded from ftp://ftp-trace.ncbi.nlm.nih.gov/giab/ftp/data/AshkenazimTrio/analysis/BCGSC_HG004_ABySS2.0_assemblies_12082016/. The GAGE-B bacterial assemblies were downloaded from https://ccb.jhu.edu/gage_b/datasets/index.html.

## Abbreviations

EvalDNA: Evaluation of De Novo Assemblies
GAGE: Genome Assembly Gold-standard Evaluations
BAM: Binary alignment map
CSA: Chromosome-sorted assembly
CH: Chinese hamster
CHO: Chinese hamster ovary
KNN: K-nearest neighbors
RF: Random forest
SVM: Support vector machines
RMSE: Root mean squared error
