# Supplementary material for: EvalDNA: a machine learning-based tool for the comprehensive evaluation of mammalian genome assembly quality
Source: BMC Bioinformatics. 2021 Nov 27;22:570. doi: 10.1186/s12859-021-04480-2 (PMC8627028; doi:10.1186/s12859-021-04480-2)
Supplement: Supplementary file 1 — Additional file 1: Additional metric descriptions, feature selection results, tables and figures. [file 12859_2021_4480_MOESM1_ESM.pdf]

Supplementary Material for ‘EvalDNA: A machine learning-based tool for the comprehensive evaluation of mammalian genome assembly quality’

Madolyn MacDonald and Kelvin Lee
September 2021

Supplementary Tables

Supplementary Table 1: Build information for each assembly used as a source of the training data chromosomes.

Organism	Recent Build (RefSeq ID)	Recent Build Release Date	Previous builds (newest - oldest)
Human	GRCh38p.12 (GCF _000001405.38)	December 2017	None Used
Human NA19420	NA19240_3.0 (GCA_001524155.4)	July 2017	NA19240_1.0
Mouse (*Mus musculus*)	GRCm38.p1 (GCF_000001635.2)	March 2012	37.2, 36.1, 35.1, 34.1, 33.1, 30
Rat (*Rattus norvegicus*)	Rnor 6.0 (GCF_000001895.5)	July 2016	Rnor 5.0 (5.1), 4.1, 3.1, 2.1

Supplementary Table 2: Summary statistics for the quality metrics selected to be included in the mammalian genome scoring model along with the reference-based quality score that the model will be estimating.

Metric	Norm. N50	Gap %	Clip	Error Free Bases	Low FC Contig	Low FC Gap	Quality Score
Min.	0.74	0.00	0.01	15.86	0.03	0.00	-79.25
1st Quantile	12.10	0.71	0.025	51.97	0.16	0.004	23.81
Median	22.77	4.04	0.032	55.34	0.59	0.23	51.83
Mean	40.16	5.43	0.08	55.73	1.60	1.65	50.55
3rd Quantile	67.12	7.567	0.06	62.02	2.35	2.17	80.68
Max.	100	43.34	0.34	68.04	15.94	11.67	100

Supplementary Table 3: Results of the Joint Mutual Information Maximization (JMIM)

algorithm from the R package Praznik.

Metric	JMIM score
error_free_bases	0.512
normN50	0.944
prop_pair_perc	0.831
links	0.758
low_fc_over_gap	0.707
gap_perc	0.627
clip	0.601
low_fc_in_contig	0.438
FCD_err_over_gap	0.358
low_read_coverage	0.231

Supplementary Table 4: %IncMSE from the importance calculation of the R package randomForest.

Metric	%IncMSE
gap_perc	172.351
low_read_cov	195.896
FCD_err_over_gap	67.259
clip	127.534
error_free_bases	310.338
Links	274.118
low_fc_over_gap	426.977
low_fc_in_contig	207.887
prop_pair_perc	68.880
normN50	256.788

Supplementary Table 5: Differences between each CH assembly EvalDNA score and PICR's EvalDNA score compared to the differences between NUCmer scores (derived from NUCmer alignments of each assembly to PICR).

Assembly	EvalDNA score	NUCmer score	Difference from PICR EvalDNA score	Difference from PICR NUCmer score
PICR	70.22	100	0	0
PIRC	70.20	99.11	0.03	0.9
IPCR	57.56	85.20	12.5	14.80
IPRC	57.57	85.24	12.64	14.76
RefSeq	58.72	85.31	11.50	14.69
CSA	43.21	63.43	27.00	36.57

Supplementary Table 6: EvalDNA scores of various Japanese rice assemblies (all three are of the

Nipponbare cultivar).

Assembly	EvalDNA score
IRGSP-1.0 (reference)	81.81
Build4.0	82.44
OrySat_Sep2003	50.82

Supplementary Table 7: EvalDNA scores and other quality metrics for human draft assemblies from the QUAST-LG study. Best values for each metric are highlighted in bold.

Assembly Metrics	UpperBound	ABYSS2	Discovar	SOAPdenovo2
**Values below do not depend on a reference assembly**				
**EvalDNA score**	**9.35**	8.78	8.42	-11.58
**Number of contigs**	**4,958**	5,014	5,802	55,725
**Total length**	2,916,500,502	2,814,649,061	2,814,592,927	3,199,541,566
**N50 contig length**	**8,821,063**	4,179,768	8,212,463	258,443
**N50 contig number**	102	197	**93**	3,296
**Number of gaps**	**33,724**	172,475	64,322	1,430,745
**Total gap length**	**3,067,652**	70,594,581	24,847,290	653,697,363
**Values below are from alignment to hg38 (http://cab.spbu.ru/files/quast/quast-lg/Human_MP/report.html)**				
**Genome fraction (%)**	**99.06**	93.56	94.81	85.10
**Total aligned length**	**2,915,987,106**	2,766,839,333	2,791,316,622	2,720,361,441
**Number of misassemblies**	**0**	820	508	670
**Misassembled contigs length**	**0**	922,999,235	1,078,600,471	164,310,662
**Number of mismatches per 100 kbp**	**0.01**	100.49	106.24	129.15
**Number of indels per 100 kbp**	**0**	27.44	25.87	50.41

Supplementary Figures

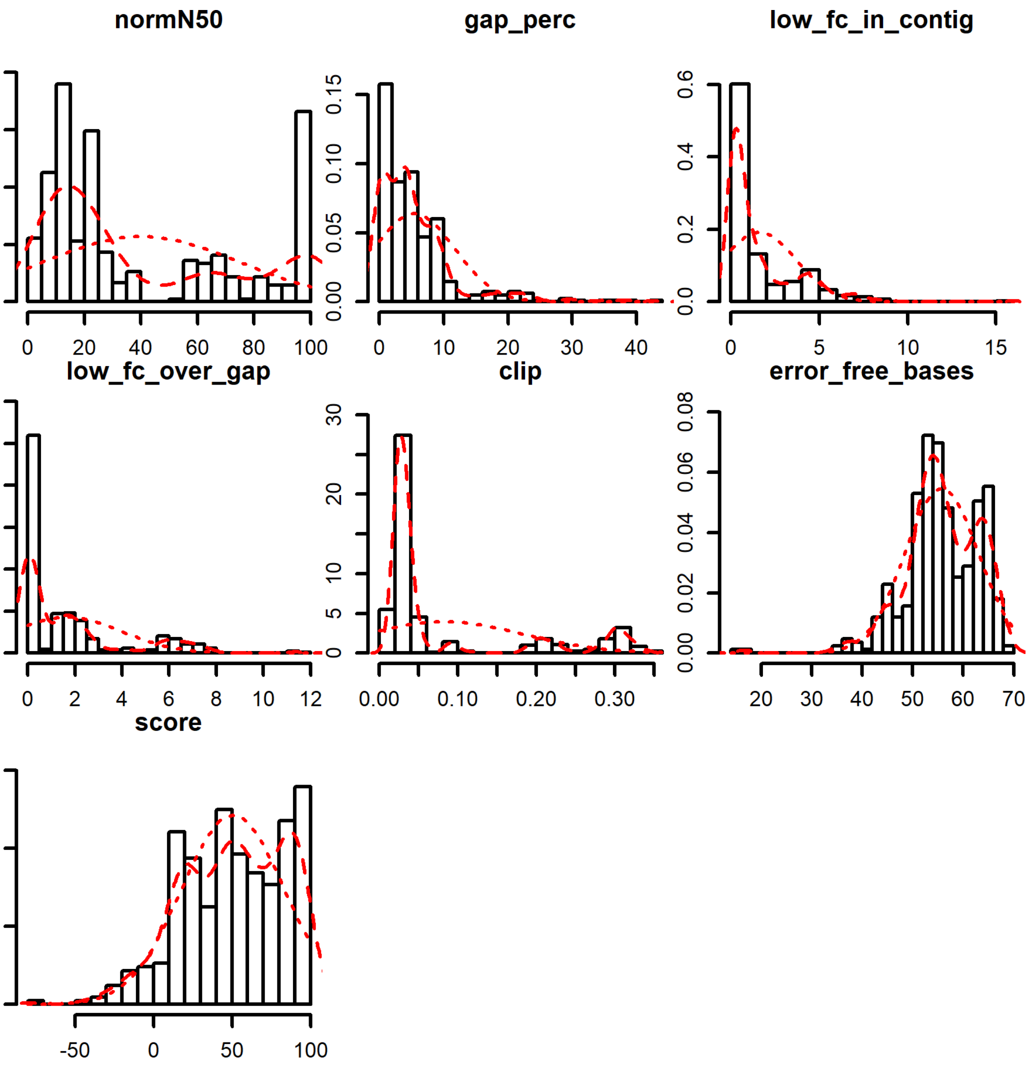

Supplementary Figure 1. Histograms for each selected genome assembly metric as well as the reference-based score (bottom left) from the training data for the mammalian model.

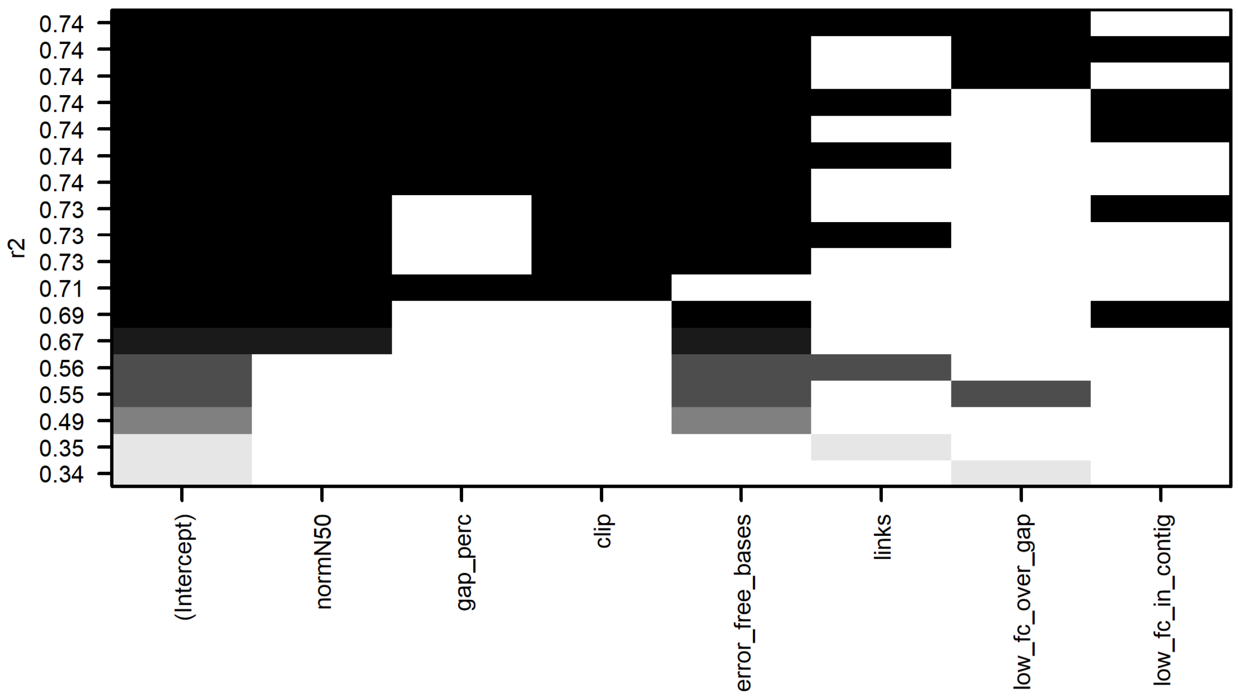

Supplementary Figure 2. Results from regsubsets function from the leaps package. Each row is a general linear model created from a subset of the features listed along the x-axis. Shaded cells indicated features included in that row's model. The models are ordered and shaded based on their r-squared value given along the y-axis.

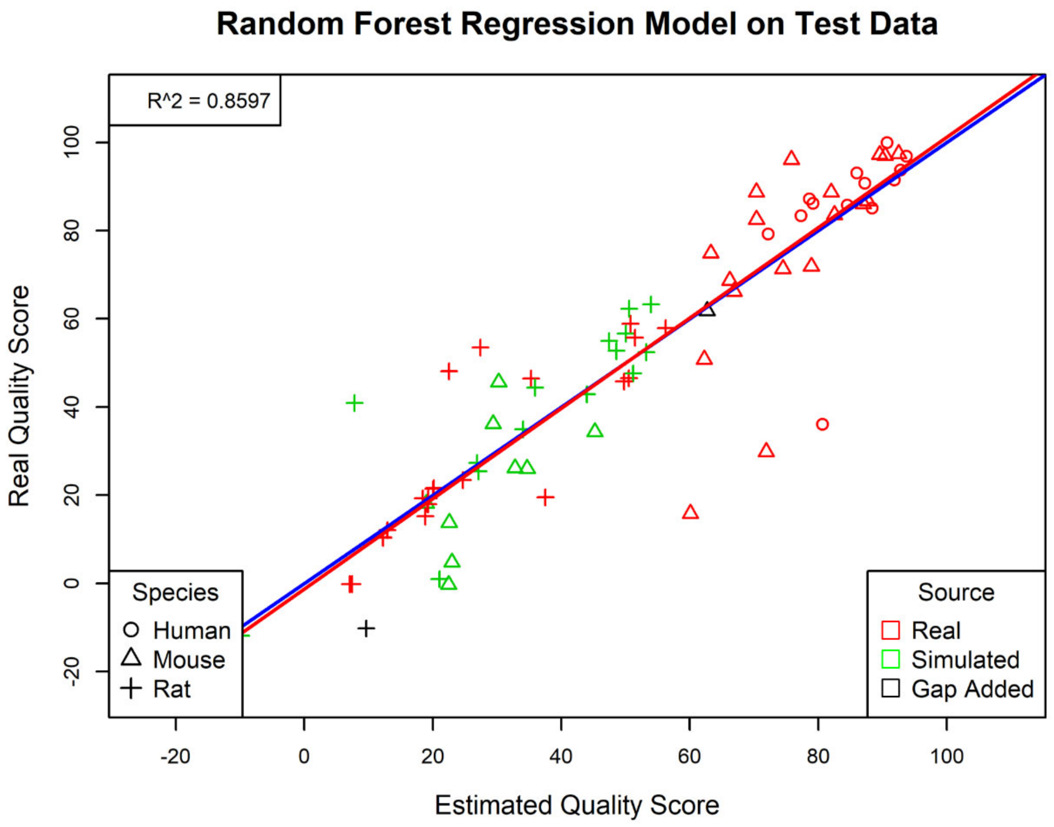

Supplementary Figure 3. Plot of the reference-based quality scores versus the EvalDNA quality scores of the test data with species (data point shapes) and sources (data point colors) denoted.

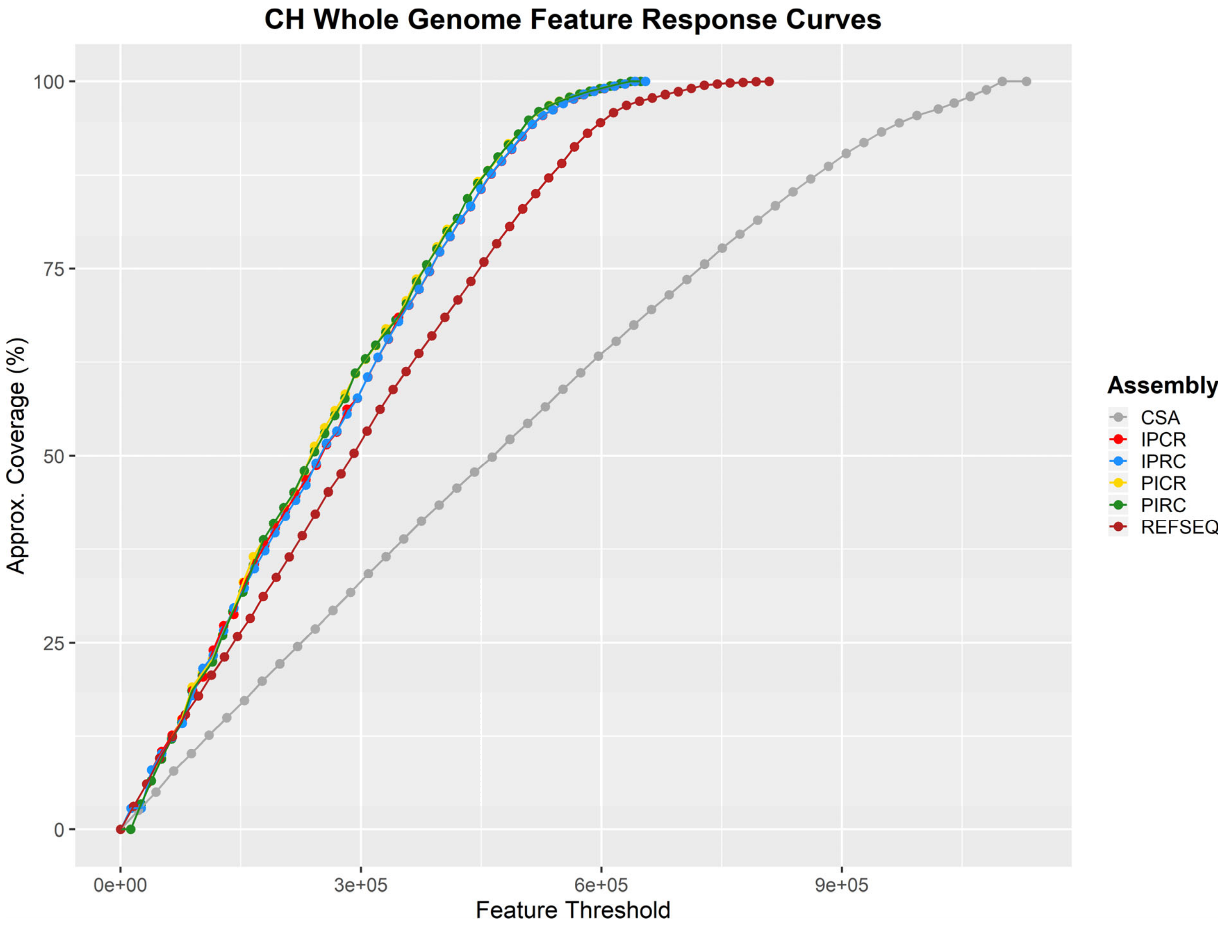

Supplementary Figure 4. FRCbam results (FRCurves) for the Chinese hamster genome assemblies. Thresholds of the number of allowed errors (features) are shown along the x-axis. Only contigs (starting with the longest) whose sum of features is less than this threshold can be used to compute the genome coverage, which is shown on the y-axis.

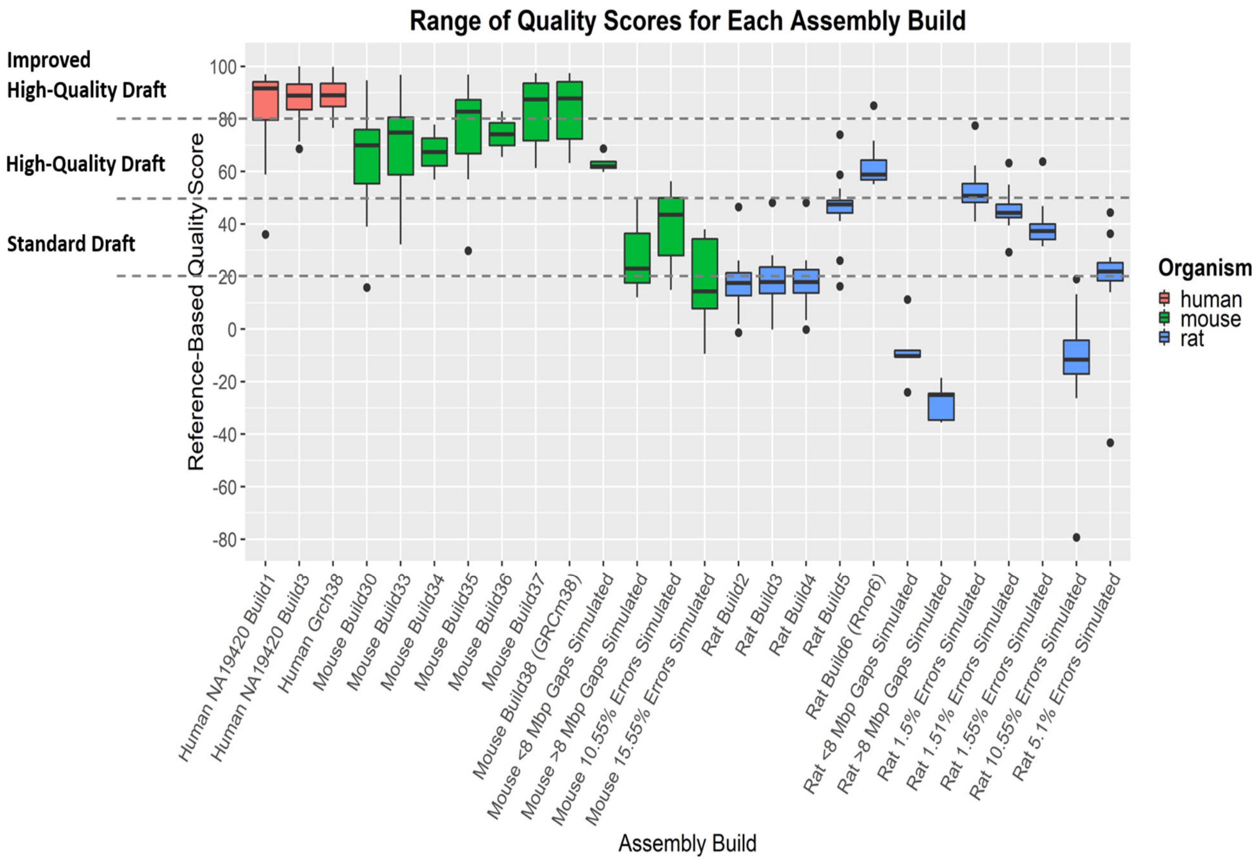

Supplementary Figure 5. Recommended guidelines for EvalDNA quality score interpretation from the reference-based scores of the training data instances.

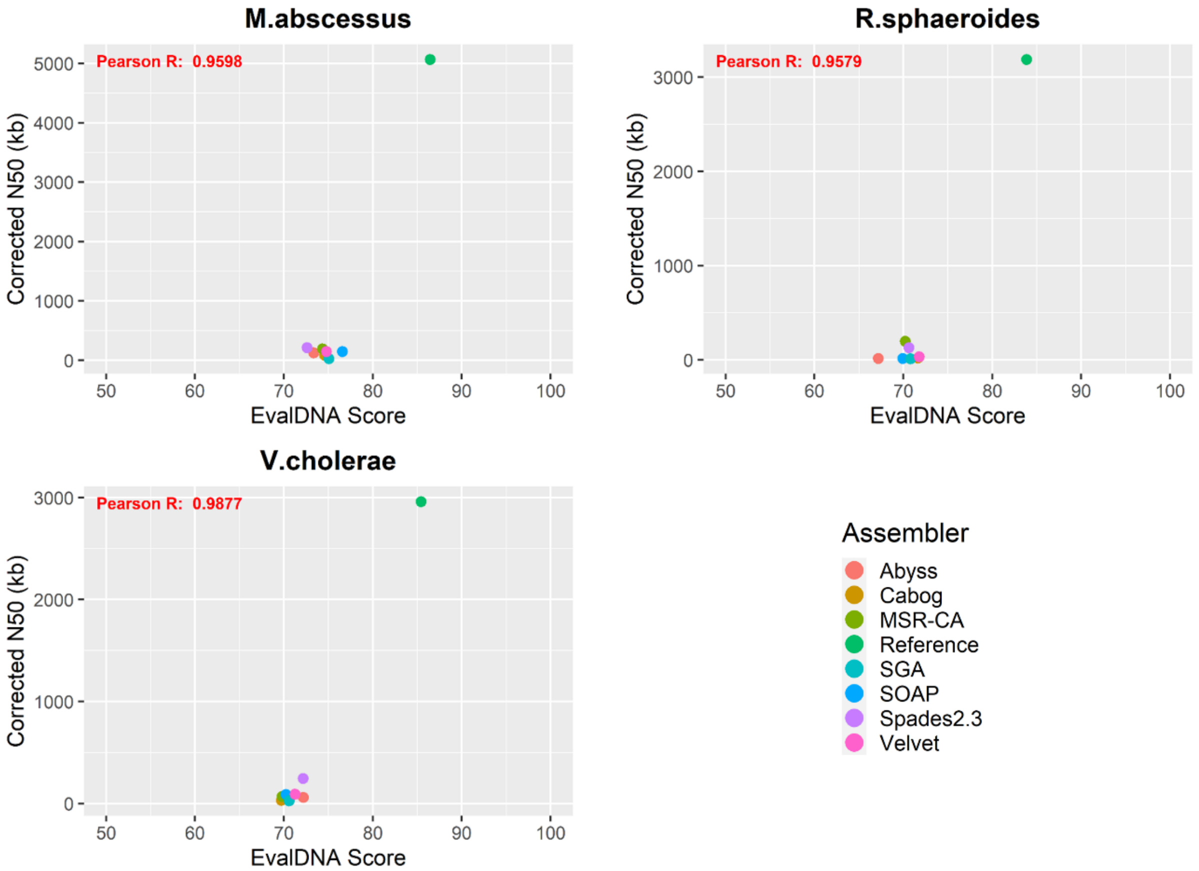

Supplementary Figure 6. The EvalDNA scores are plotted against the corrected N50 of bacteria assemblies from the GAGE-B study that were created using different assemblers. The Pearson correlation coefficient is provided in red.

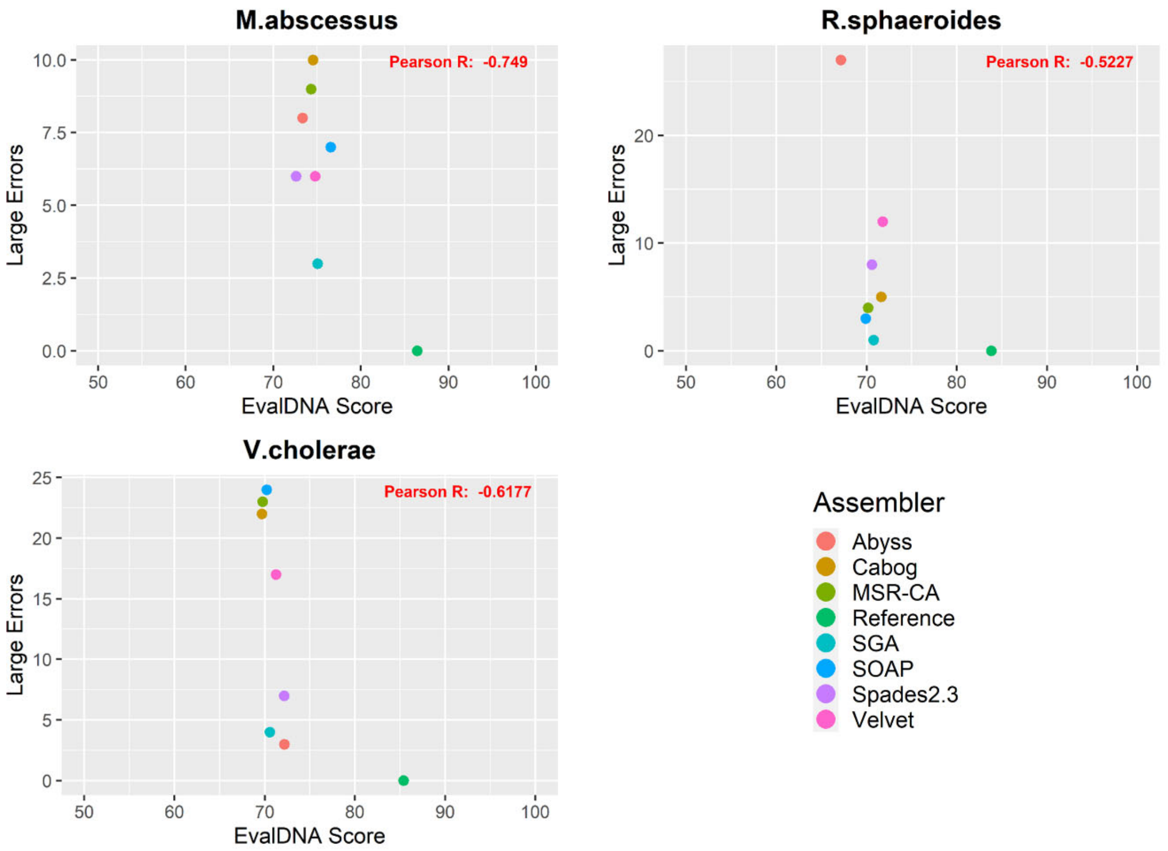

Supplementary Figure 7. The EvalDNA scores are plotted against the amount of large errors in the bacteria assemblies from the GAGE-B study that were created using different assemblers. The Pearson correlation coefficient is provided in red.

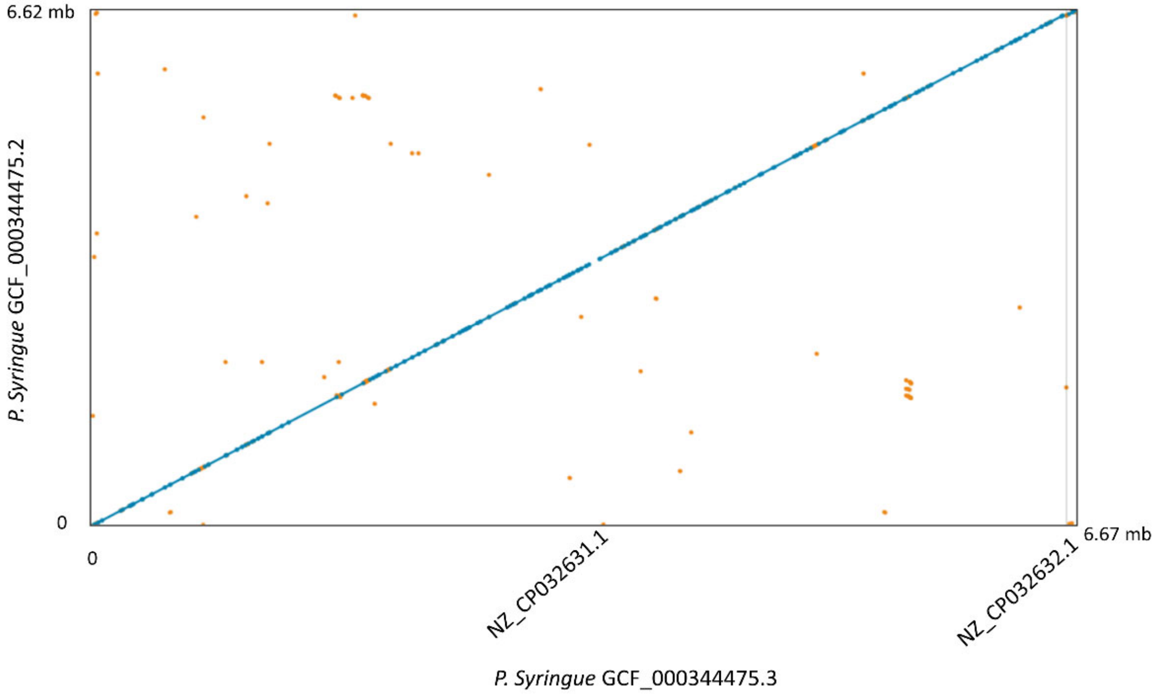

Supplementary Figure 8. Alignment of two versions of the *P. syringue* (strain Shaanxi MG228) genome assembly using NUCmer. For a more interpretable visualization, the contigs in the GCF_000344475.2 assembly were merged based on the gaps and overlaps in the original assembly alignment.

Quality Metric Definitions

All quality metrics examined are described here. Metrics are converted into percentage of bases per assembled sequence or normalized by assembly length if needed. Note that not all of the metrics were included in the final model due to multicollinearity or lack of significant correlation with the reference-based quality score in the training data.

1. Normalized N50 length - N50 length normalized by total sequence length.

$$normN50 = \frac{N50\_length}{total\_length} x\ 100, \qquad 0 \leq N50\_length\ \leq 100$$

2. Gap percent - percent of total bases which are gaps (N's).

$$gap\_percent = \frac{total\_gap\_length}{total\_length} x\ 100, \qquad 0 \leq gap\_percent \leq 100$$

3. Normalized contig count - Number of separate pieces (scaffolds/contigs) the sequence of interest is split into normalized by the sequence length in megabases (Mbp).

$$norm\_contig = \frac{number\_of\_contigs}{total\_length} x\ 100{,}000{,}000, \qquad 0 \leq norm\_contig \leq 100{,}000{,}000$$

4. Links - percent of total bases impacted by link errors called by REAPR. These bases are located in regions where a significant proportion of the reads mapped to this region also mapped elsewhere.

$$links = \frac{number\_of\_bases\_in\_link\_regions}{total\_length} x\ 100, \qquad 0 \leq links \leq 100$$

5. Collapsed repeats - percent of total bases impacted by the collapsed repeat errors called by REAPR.

$$collapsed\_repeats = \frac{number\_of\_bases\_in\_collapsed\_repeat\_regions}{total\_length} x\ 100, \qquad 0 \leq collapsed\_repeats \leq 100$$

6. Clip - percent of total bases impacted by the clip errors called by REAPR. These bases are located in regions where a significant proportion of the reads had to be clipped to map to this region.

$$clip = \frac{number\_of\_bases\_in\_clip\_regions}{total\_length} x\ 100, \qquad 0 \leq clip \leq 100$$

7. Low read coverage - percent of total bases impacted by the low read coverage errors called by REAPR. These bases are in regions with low coverage of proper paired reads.

$$low\_read\_coverage = \frac{number\_of\_bases\_in\_low\_read\_coverage\_regions}{total\_length} x\ 100, \qquad 0 \leq low\_read\_coverage \leq 100$$

8. Properly paired read percent - percent of mapped reads that are properly paired as determined by SAMtools.

$$proper\_pair\_percent = \frac{total\_reads\_in\_proper\_pairs}{total\_reads\_mapped} x\ 100, \qquad 0 \leq proper\_pair\_percent \leq 100$$

9. Error free bases - percent of bases called by REAPR as error free. A base is called error free if it has at least 5x coverage of perfect and unique mapped reads.

$$error\_free\_bases = \frac{number\_of\_error\_free\_bases}{total\_length} x\ 100, \qquad 0 \leq error\_free\_bases \leq 100$$

10. Fragment coverage distribution (FCD) errors in contig - percent of bases in regions that REAPR marks as an FCD error within a contig (the region does not contain any gaps).

$$FCD\_err\_in\_contig = \frac{number\_of\_bases\_in\_FCD\_error\_contig\_regions}{total\_length} x\ 100, \qquad 0 \leq FCD\_err\_in\_contig \leq 1$$

11. FCD errors over gap - percent of bases in regions that REAPR marks as an FCD error and the region contains a gap.

$$FCD\_err\_over\_gap = \frac{number\_of\_bases\_in\_FCD\_error\_regions\_with\_gap}{total\_length} x\ 100, \qquad 0 \le FCD\_err\_over\_gap \le 100$$

12. Low fragment coverage (FC) in contig - percent of bases in regions that REAPR marks as having low fragment coverage and the region does not contain any gaps.

$$low\_fc\_in\_contig = \frac{number\_of\_bases\_in\_low\_FC\_contig\_regions}{total\_length} x\ 100, \qquad 0 \le low\_fc\_in\_contig \le 1$$

13. Low fragment coverage (FC) over gap - percent of bases in regions that REAPR marks as having low fragment coverage and the region contains a gap.

$$low\_fc\_over\_gap = \frac{number\_of\_bases\_in\_low\_FC\_regions\_with\_gap}{total\_length} x\ 100, \qquad 0 \le low\_fc\_over\_gap \le 100$$

Model Testing

Information about the other models that were examined as well as their RMSE and R-squared values are provided in this section.

1. General linear model with scaling

   (a) Model:
   quality score = 51.531 + normN50 * 15.450 + gap perc * -8.267 + clip * -11.175 + error free bases * 8.058 + low fc over gap * -1.267 + low fc in contig * -2.187

   (b) Results on test data:
   RMSE = 16.413
   R-Squared = 0.775

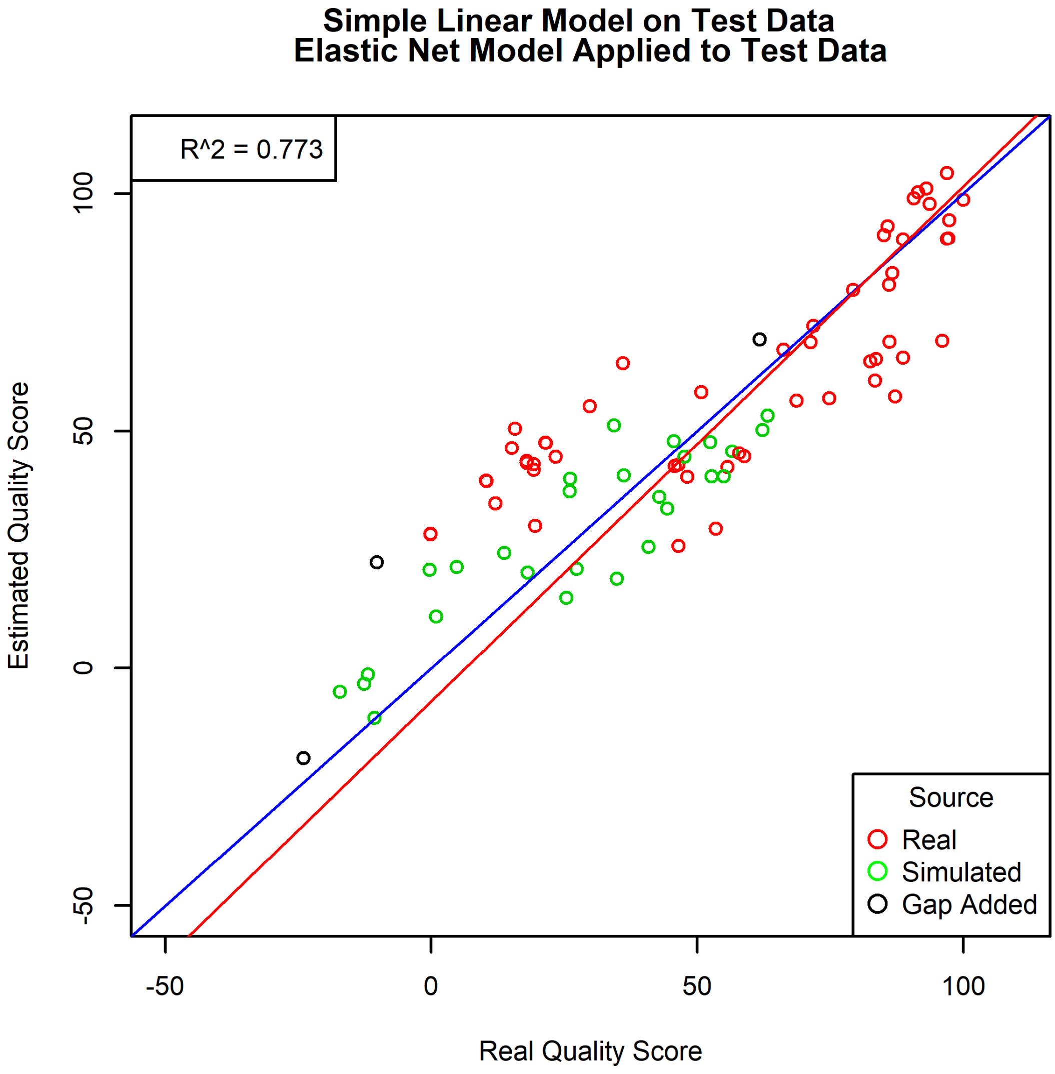

Supplementary Figure 6. Performance of the general linear regression model on test data. The estimated quality scores of the test instances are plotted against the reference-based quality scores of the test instances.

2. Elastic Net - Caret train method 'glmnet' with scaling
   (a) Model:
   alpha = 0.1
   lambda = 0.677
   quality score = 51.531 + normN50 * 14.939 + gap perc * -7.689 + clip * -10.459 + error free bases * 8.353 + low fc over gap * -1.501 + low fc in contig * -2.330
   (b) Results on test data:
   RMSE = 16.520
   R-square = 0.773

Supplementary Figure 7. Performance of the elastic net regression model on test data. The

estimated quality scores are plotted against the reference-based quality scores of the test instances.

3. K-Nearest Neighbors (KNN) regression

(a) Tuning parameters

RMSE was used to select the model with the most optimal k value. The final value used for the model was k = 5.

Supplementary Table 7: Results for tuning the value of k for KNN regression.

k	RMSE	R-squared	MAE
5	11.753	0.873	8.459
7	12.006	0.869	8.598
9	12.463	0.860	8.922

(b) Results on test data:

RMSE = 13.615
R-square = 0.840

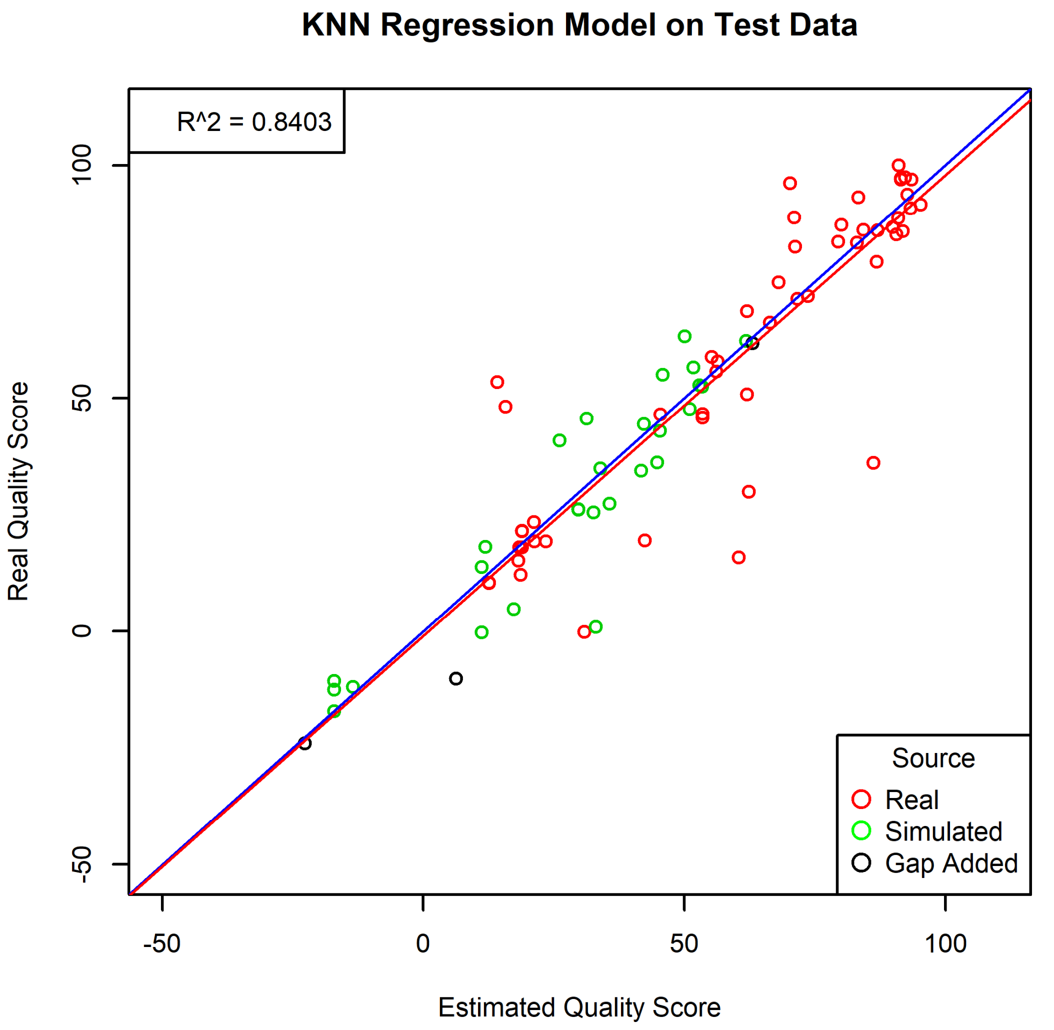

Supplementary Figure 8. Performance of the KNN regression model on test data. The estimated quality scores are plotted against the reference-based quality scores of the test instances.

4. Support Vector Machines with Linear Kernel

   (a) Tuning parameters 'C' was held constant at a value of 1

   (b) Results on Test Data:

   RMSE = 17.190
   R-square = 0.774

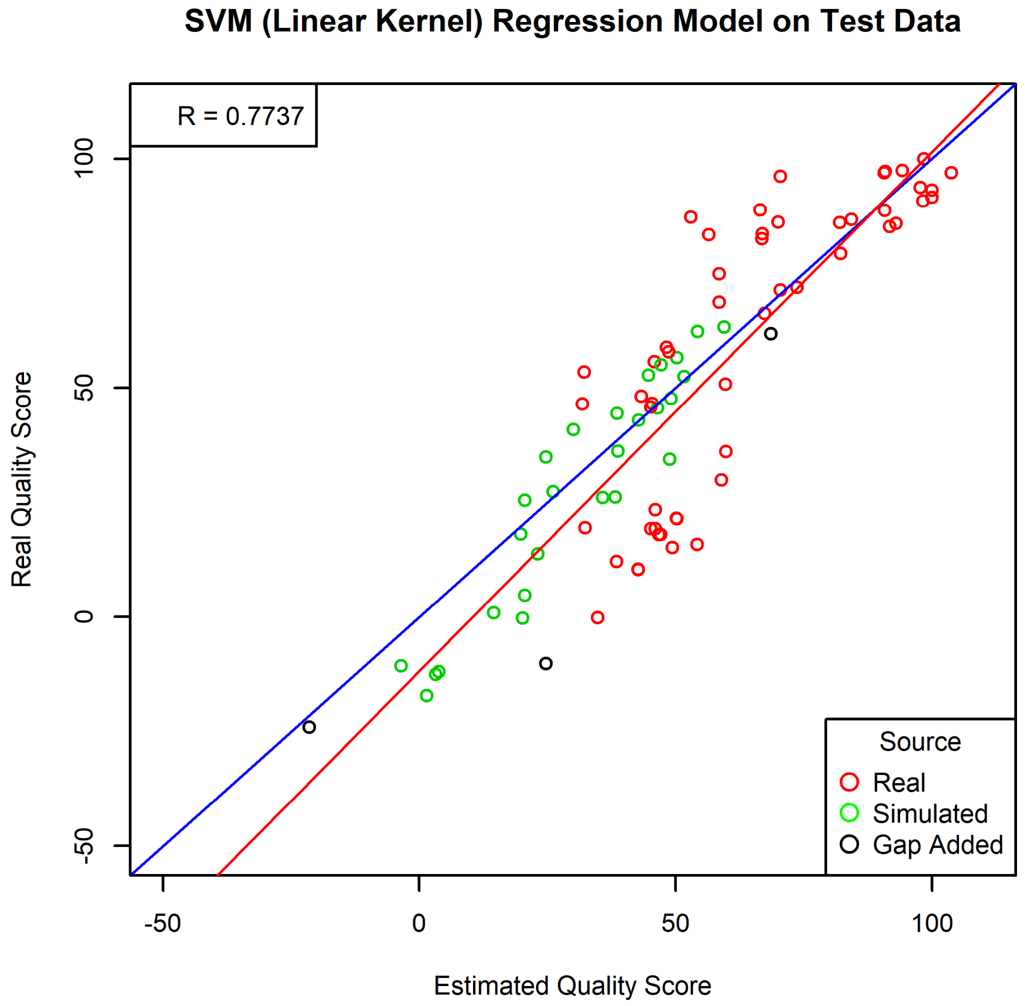

Supplementary Figure 10. Performance of the SVM regression model with a linear kernel on test data. The estimated quality scores are plotted against the reference-based quality scores of the test instances.

5. Support Vector Machines (SVM) regression with Polynomial Kernel

   (a) Tuning parameters:

   RMSE was used to select the optimal value of C, degree, and scale for the Polynomial Kernel SVM. The final values used for the model were degree = 2, scale = 0.1 and C = 0.25.

Supplementary Table 8: Results for tuning the value of C for SVM regression with a polynomial basis function kernel.

Degree	Scale	C	RMSE	R-squared	MAE
1	0.001	0.25	29.499	0.646	24.060
1	0.001	0.50	26.973	0.652	21.646
1	0.001	1.00	24.083	0.670	18.797
1	0.010	0.25	20.392	0.694	15.301
1	0.010	0.50	18.777	0.709	13.829
1	0.010	1.00	18.045	0.715	13.306
1	0.100	0.25	17.470	0.721	12.947
1	0.100	0.50	17.408	0.719	12.853
1	0.100	1.00	17.406	0.718	12.802
2	0.001	0.25	26.966	0.652	21.641
2	0.001	0.50	24.073	0.670	18.789
2	0.001	1.00	21.103	0.688	15.963
2	0.010	0.25	18.558	0.716	13.672
2	0.010	0.50	17.585	0.728	13.017
2	0.010	1.00	16.854	0.743	12.477
2	0.100	0.25	16.039	0.791	11.802
2	0.100	0.50	16.466	0.792	11.842
2	0.100	1.00	16.288	0.794	11.639
3	0.001	0.25	25.233	0.662	19.923
3	0.001	0.50	22.089	0.682	16.894
3	0.001	1.00	19.880	0.699	14.798
3	0.010	0.25	17.643	0.731	13.050
3	0.010	0.50	16.777	0.749	12.363
3	0.010	1.00	16.079	0.769	11.939
3	0.100	0.25	19.824	0.778	11.562
3	0.100	0.50	20.101	0.788	11.267
3	0.100	1.00	18.799	0.800	10.645

(b) Results on test data:

RMSE = 14.363
R-square = 0.843

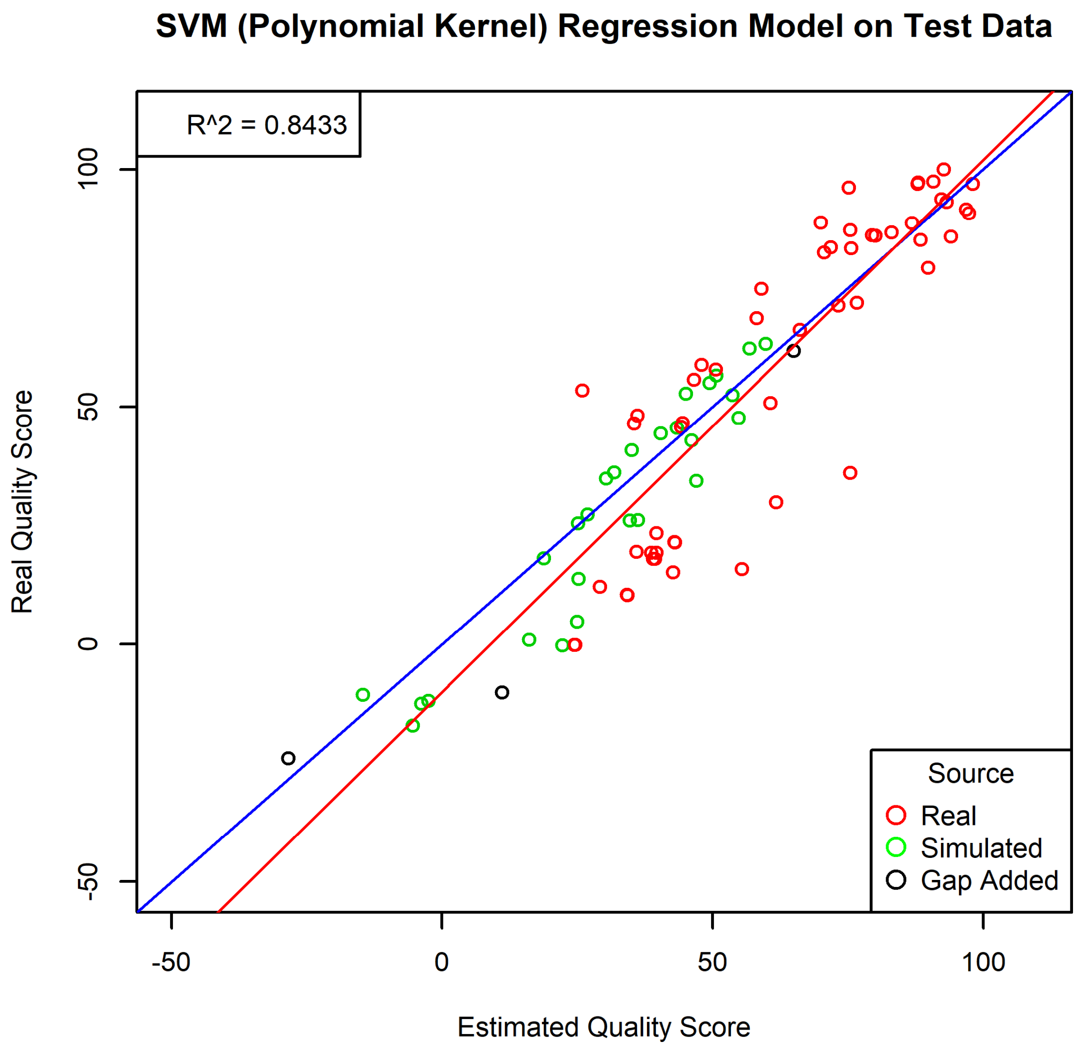

Supplementary Figure 9. Performance of the SVM regression model with a polynomial kernel on test data. The estimated quality scores are plotted against the reference-based quality scores of the test instances.

Model without the normalized N50 metric

A random forest regression model using the same metrics as the main mammalian model, except for normN50, was developed. Parameters were tuned for using 10-fold cross validation. The lowest value of RMSE was used to select the best value of mtry, which was mtry = 3 (Table 9). The model was applied to test data and produced a R-squared value of 0.817 and an RMSE of 14.483.

Supplementary Table 9: Results for tuning the value of mtry for random forest regression model (500 trees).

mtry	RMSE	R-squared	MAE
2	12.758	0.852	9.404
3	12.554	0.855	9.181
5	12.793	0.852	9.130

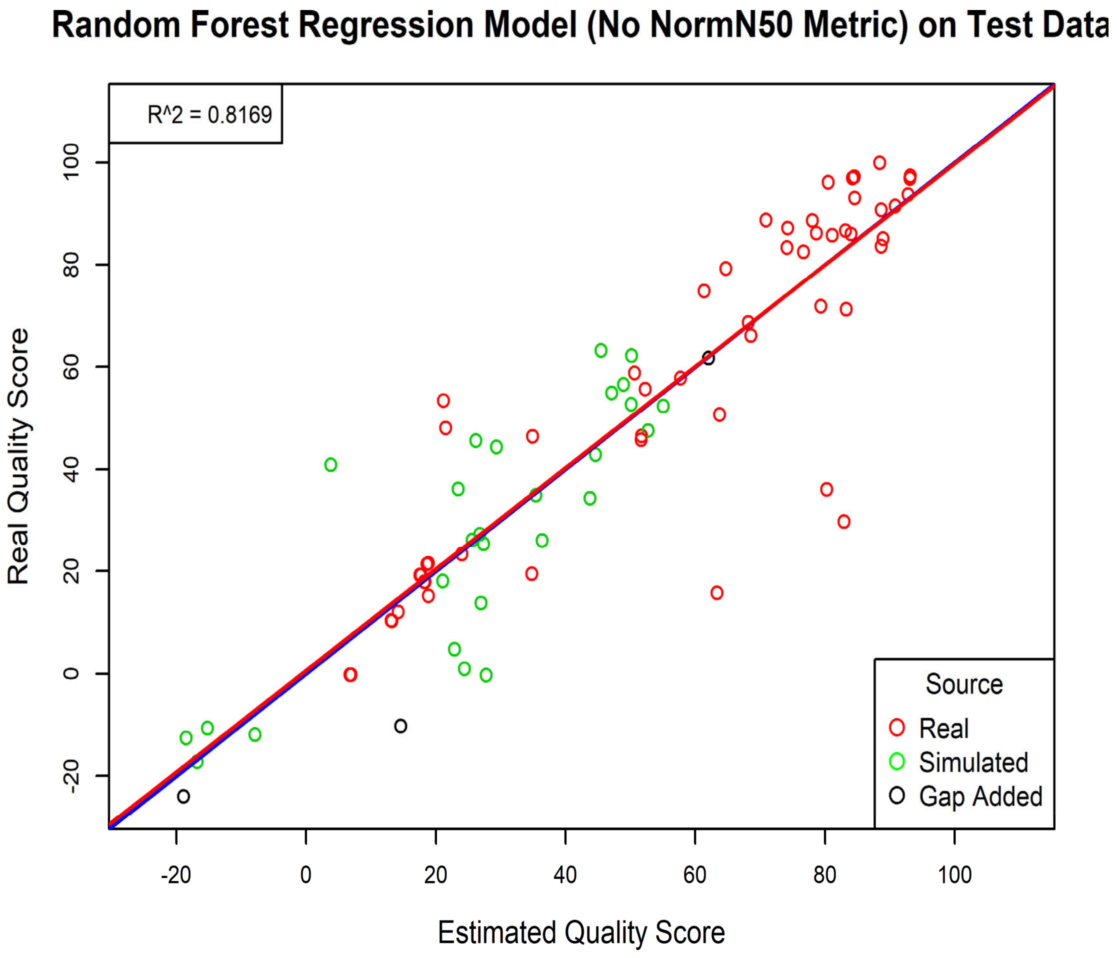

Supplementary Figure 11. Performance of the random forest regression model without the normN50 metric on test data. The estimated quality scores are plotted against the reference-based quality scores of the test instances. A 100% accurate model would produce the blue line with an r-squared equal to 1. The line of best fit for the plotted data is shown as the red line and has an r-squared of 0.8169.
